# The Ontogenetic Osteohistology of *Tenontosaurus tilletti*

**DOI:** 10.1371/journal.pone.0033539

**Published:** 2012-03-28

**Authors:** Sarah Werning

**Affiliations:** Department of Zoology and Sam Noble Oklahoma Museum of Natural History, University of Oklahoma, Norman, Oklahoma, United States of America; Raymond M. Alf Museum of Paleontology, United States of America

## Abstract

*Tenontosaurus tilletti* is an ornithopod dinosaur known from the Early Cretaceous (Aptian-Albian) Cloverly and Antlers formations of the Western United States. It is represented by a large number of specimens spanning a number of ontogenetic stages, and these specimens have been collected across a wide geographic range (from central Montana to southern Oklahoma). Here I describe the long bone histology of *T. tilletti* and discuss histological variation at the individual, ontogenetic and geographic levels. The ontogenetic pattern of bone histology in *T. tilletti* is similar to that of other dinosaurs, reflecting extremely rapid growth early in life, and sustained rapid growth through sub-adult ontogeny. But unlike other iguanodontians, this dinosaur shows an extended multi-year period of slow growth as skeletal maturity approached. Evidence of termination of growth (e.g., an external fundamental system) is observed in only the largest individuals, although other histological signals in only slightly smaller specimens suggest a substantial slowing of growth later in life. Histological differences in the amount of remodeling and the number of lines of arrested growth varied among elements within individuals, but bone histology was conservative across sampled individuals of the species, despite known paleoenvironmental differences between the Antlers and Cloverly formations. The bone histology of *T. tilletti* indicates a much slower growth trajectory than observed for other iguanodontians (e.g., hadrosaurids), suggesting that those taxa reached much larger sizes than *Tenontosaurus* in a shorter time.

## Introduction

*Tenontosaurus tilletti* is an ornithopod dinosaur known from the Early Cretaceous of the western United States. Numerous specimens, including many partial and nearly complete skeletons, have been collected across a wide geographic range. Ostrom [1] first described *T. tilletti* based on specimens collected from multiple localities in the Cloverly Formation exposed across northern Wyoming and central Montana. Sues and Norman [2] and Brinkman et al. [3] later described specimens from the Antlers Formation of southern Oklahoma, although specimens were known from this formation at the time of Ostrom's original work [1]. Winkler [4] additionally referred a specimen from the Paluxy Formation of northern Texas to *T. tilletti*. Today, *Tenontosaurus* is well-sampled from both the Antlers and Cloverly formations; there are over thirty partial or complete skeletons in North American museum collections. Additional materials have been assigned to *T. tilletti* from the Arundel Formation of Maryland [5], the Cedar Mountain Formation of Utah [6], the Paluxy and Twin Mountains formations of Texas [4], [7], [8], the Wayan Group of Idaho [9], and the Shellenberger Canyon Formation of Arizona [5], [10], but with the exception of the Paluxy specimens, these materials either have been shown to represent other taxa (*T. dossi* in the Twin Mountains Formation [4], [8]) or are not diagnostic (e.g., teeth, isolated femur or ribs). Regardless, the known paleogeographic distribution of *T. tilletti* is extensive compared to most dinosaur species.

*Tenontosaurus* is also well-sampled ontogenetically; Ostrom [1] noted that most of the available material was from juvenile and sub-adult individuals. Ontogenetic changes in the postcranial morphology of *T. tilletti* were described by Forster [11], and specimens ranging from very small juveniles (possible neonates) to large adults have been collected from across its paleogeographic range [1], [3], [11], [12], allowing confidence in identification and taxonomic assignment of elements through ontogeny.

**Figure 1:**
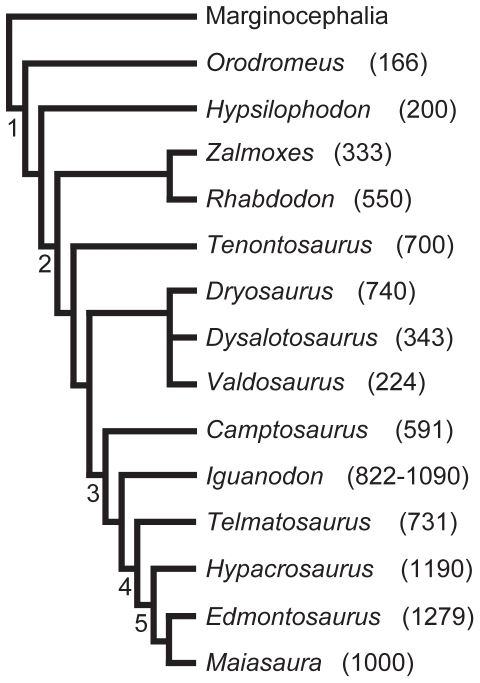
Phylogenetic relationships and adult femoral lengths (mm) of ornithopod taxa discussed in this study. This tree is pruned from recently published estimations of ornithischian [14] and ornithopod [15], [82] dinosaurs. These studies do not conflict in their assessment of relationships of these taxa. Node numbers correspond to the following clades: 1 – Ornithopoda, 2 – Iguanodontia, 3 – Ankylopollexia, 4 – Hadrosauroidea, 5 – Hadrosauridae. Femoral lengths are taken from [81], except *Zalmoxes* [83] and *Maiasaura* [17].

Previous studies found *Tenontosaurus* to be the sister taxon to all other iguanodontians [13]. However, more recent analyses have recovered *Tenontosaurus* and Rhabdodontidae (*Muttaburrasaurus*, *Rhabdodon*, and *Zalmoxes*) as the successive sister taxa to Iguanodontia [14], [15] (also see Figure 1), a diverse clade that includes all of the large-bodied ornithopods (e.g., *Iguanodon*, hadrosaurs). Hadrosaurs are characterized by extremely high growth rates, and reached their large adult mass in a decade or less [16]–[19]. Non-iguanodontian ornithopods are almost exclusively small- and medium-bodied taxa comparable in size to *Tenontosaurus* or smaller (see Figure 1 for femoral lengths of ornithopods discussed in this study). Given its phylogenetic position, *Tenontosaurus* may serve as a “baseline” or outgroup condition for growth and histology for the rest of Iguanodontia, and for large-bodied ornithopods in general. Because there are many available specimens from all stages of ontogeny and a broad geographic distribution, and because the taxon is intermediate in both body size and phylogenetic position between the “hypsilophodontids” and hadrosaurids, *Tenontosaurus* is important for understanding the relationship between ornithopod body size and growth, and potentially offers clues to the origin and evolution of hadrosaurian growth rates.

There are three goals to my study. First, I will describe the ontogenetic changes in the osteohistology of the main limb bones of *Tenontosaurus*. Second, I will describe and compare the amount and nature of histovariability observable at the individual (within-skeleton), ontogenetic, and population (geographic) levels. Finally, I will compare the ontogenetic osteohistology of *Tenontosaurus* to that of other ornithopods in phylogenetic context, in order to elucidate phylogenetic trends in histology and growth dynamics.

## Materials and Methods

### Institutional abbreviations

The Field Museum, Chicago, Illinois (FMNH); Museum of the Rockies, Bozeman, Montana (MOR); Sam Noble Oklahoma Museum of Natural History, Norman, Oklahoma (OMNH); Southern Methodist University, Dallas, Texas (SMU); Vertebrate Paleontology Laboratory, University of Texas at Austin, Austin, Texas (TMM); University of California Museum of Paleontology, Berkeley, California (UCMP)

### Note on methods

I wrote this methods section in consultation with a fossil preparator (M. Brown, personal communication), in order to craft a level of description that would allow future researchers to reproduce my methods accurately. Preparation (and imaging) methods can directly affect interpretations of fossil morphological data [20]. Because my entire histological dataset is available to researchers as digital images for future analysis, it is essential to report any preparation methods that could affect their interpretation or analysis.

### Slide preparation

I sampled the mid-diaphysis of long bones (humerus, ulna, femur, tibia, and fibula) from individuals of *Tenontosaurus tilletti* in the FMNH and OMNH collections representing a range of sizes spanning ontogeny (see Table 1 for a list of specimens sampled). I chose specimens based on their size, preservation, and association with diagnostic elements. These long bones are the most commonly preserved elements for *Tenontosaurus*, experience the least change in the external morphology of the diaphysis during ontogeny, and comprise the main weight-bearing bones during quadrupedal locomotion. I also examined and imaged specimens from the MOR produced for another osteohistological study [18]; see that publication for the details of those slides' preparation.

**Table 1:** List of *Tenontosaurus* specimens sampled and examined for this study.

Specimen	Formation	Age	Elements sampled
MOR 788	Cloverly	perinate	tibia*
FMNH PR 2263	Cloverly	juvenile	femur
MOR 679	Cloverly	juvenile	femur*
OMNH 10144	Cloverly	juvenile	humerus, ulna, femur, tibia
OMNH 34785	Cloverly	juvenile	femur, tibia, fibula
MOR 682	Cloverly	subadult	femur*
OMNH 2531	Antlers	subadult	humerus, ulna
OMNH 2926	Antlers	subadult	tibia
OMNH 8137	Antlers	subadult	humerus, tibia
OMNH 10134	Cloverly	subadult	tibia
OMNH 16563	Antlers	subadult	tibia, fibula, rib
OMNH 34191	Antlers	subadult	ulna
OMNH 34783	Cloverly	subadult	tibia, fibula
OMNH 34784	Cloverly	subadult	femur, tibia
OMNH 58340	Antlers	subadult	tibia
OMNH 63525	Antlers	subadult	tibia, rib
FMNH PR 2261	Cloverly	adult	humerus, ulna, femur, tibia
OMNH 62990	Antlers	adult	ulna

Specimens indicated with an asterisk were histologically sampled as part of a previous study [17], but were re-examined and re-imaged for this project.

The long bones of the OMNH and FMNH specimens had been mechanically prepared (i.e., the matrix was removed from external surfaces) with pneumatic and hand tools prior to my study. Before sampling, I visually inspected each bone and no external surface damage resulting from preparation (e.g., tool marks, abrasion) was apparent on the mid-diaphyses of these specimens. However, it is possible that the outermost cortex and/or bone surface may have been locally damaged during preparation. I measured and photographed each element, removed a portion of the mid-diaphysis along natural breaks, and molded and cast each removed section to preserve the morphological information lost through destructive sampling [21]. Juvenile elements were molded and cast in entirety before removing the mid-diaphyseal section. Several molding agents were used during this project; these include Silputty platinum-based quick curing room-temperature vulcanizing silicone rubber (Silpak, Inc., North Hollywood, California); GT-5092 condensation cure silicone rubber base catalyzed with CA-5275 Fast Catalyst silicone curing agent (GT Products, Inc., Grapevine, Texas); or Rhodorsil V-1065 condensation cure silicone rubber base catalyzed with Hi-Pro Green catalyst (Rhodia; now Bluestar Silicones, East Brunswick, New Jersey). All specimens were cast in TC-881 A/B or TC-891 A/B rigid polyurethane casting resin (BJB Enterprises, Inc., Tustin, California), pigmented neutral grey with a mix of black (6836) and white (6834) liquid pigments (BJB Enterprises, Inc.). All molds and casts are reposited at the home institution of the specimens sampled (FMNH, OMNH).

I produced my histological thin-sections using standard fossil histology techniques (e.g., [22], [23]), with the following chemical/equipment modifications. I first cleaned the surfaces of the mid-diaphyseal sections with acetone in order to remove any glues/stabilizers from the bone surface and to allow better penetration of the embedding resin. After drying, I embedded all bone sections in Silmar-41 clear polyester casting resin (Interplastic Corporation, Saint Paul, Minnesota) catalyzed with methyl ethyl ketone peroxide (Norac, Inc., Helena, Arkansas) at .7–1% by mass and allowed them to cure for 24–72+ hours before sectioning. I heated all embedded specimens that cured less than 48 hours in an oven for one hour at 50–60°C to drive the curing reaction to completion, and then allowed them to cool completely before sectioning. No specimen was allowed to cure for less than 24 hours before heating/sectioning. I cut thick sections of between 1 and 8 mm in thickness from each embedded specimen using a diamond-tipped wafering blade on a low-speed Isomet lapidary saw (Buehler, Inc., Lake Bluff, Illinois) or a diamond-tipped blade on a large tile saw (multiple brands), depending on the size of the specimen. I wet-ground the mounting-side of these sections manually using 600/P1200-grit BuehlerMet II abrasive papers (Buehler, Inc.) and an EcoMet3 or EcoMet 4000 grinder/polisher (Buehler, Inc.). I mounted the resulting samples to glass slides using water clear 2-ton epoxy (Devcon, Danvers, Massachusetts) and allowed them to cure for at least 24 hours before grinding. I manually wet-ground the slides using CarbiMet, CarbiMet 2, or BuehlerMet II abrasive papers of increasing grit size (60/P60, 120/P120, 180/P180, 320/P400, 400/P800, 600/P1200, 1200/P2500) until a final thickness of ∼30–60 microns, then polished the slides using 5 micron aluminum oxide abrasive powder (Buehler, Inc.) and a MicroCloth synthetic polishing cloth (Buehler, Inc.). Some specimens were further polished with 1 micron MicroPolish II deagglomerated alumina powder (Buehler, Inc.).

### Imaging and image analysis

I examined each slide under regular transmitted light and using crossed Nicols (full wave tint plate, λ = 530 nm) and crossed plane-polarizing filters. The filters were used to enhance birefringence.

For all specimens except OMNH 10144 (humerus, ulna, and tibia only) and OMNH 34785 (fibula only), images of the entire mid-diaphyseal cross-section were taken using a DS-Fi1 digital sight camera (Nikon Inc., Melville, New York) mounted to an Optiphot-Pol light transmission microscope (Nikon Inc.). Images were taken at 4× total magnification, 1280×960 resolution, and at uniform contrast. These cross-sectional images were taken across the entire sample at 15% overlap (both X and Y directions using the automated stage) and assembled using NIS-Elements Basic Research 3.0 (Nikon Inc.). For some very large samples, or cases in which specimen thickness was not even across the entire sample, water or a small amount of baby oil (Johnson & Johnson, New Brunswick, New Jersey) was used to increase the refraction index for clarity during photography. Further processing of these images (e.g., to adjust brightness or contrast, and/or to add text and scale bars) was completed using Photoshop CS5 Extended (Adobe Systems Inc., San Jose, California).

For the cross-sectional images of the specimens/elements listed above, overlapping digital images photographs (overlap 50% by eye in X and Y directions) were taken under regular transmitted light at 5× total magnification using a D300 DSLR camera (Nikon Inc.) through an Optiphot2-Pol light transmission microscope (Nikon Inc.). These digital photomicrographs were taken as 8-bit jpgs (quality = fine, compression = optimal quality, image size = large/4288×2848 pxl). The interface program for these images was Camera Control Pro 2 (Nikon Inc.) running on a Windows 7 (64-bit; Microsoft, Redmond, Washington) computer (HP, Palo Alto, California). I then assembled photomontages of the full cross-sections using Autopano Giga 2.0 64Bit (Kolor, Challes-les-Eaux, France), with the following settings: Detection settings: detection quality = high, layout = free. Optimization settings: strong (for partial cross-sections) and gigapixel (for full cross-sections), optimizer stages: local approach, strong algorithm, first optimization, clean up control points or links, keep only control points below the error RMS = 2.0, final optimization, advanced distortion. Panorama settings: preferred projection = automatic, preferred extend = clamp to panorama content, initial type of anchor = mono transfer function. Render settings: size = 100%, algorithms: interpolating = bicubic, blending = smartblend, format = jpg, depth = 8 bits, layers = none, DPI = 72.

Additional, detailed histological photomicrographs of some FMNH and OMNH specimens were taken at 10×, 25× and/or 100× total magnification. Some of these were montaged using Autopano Giga and the above settings. All images were then adjusted using Photoshop CS5 Extended for brightness and contrast and to add text and scale bars. At full resolution, the scale conversions for the images taken with the D300 are as follows: 5× (including montages): 925 pxl = 1.0000 mm, 10×: 1850 pxl = 1.0000 mm, 25× (including montages): 4625 pxl = 1.0000 mm, 100×: 1850 pxl = .1000 mm. Further processing of montaged images (e.g., to adjusting brightness or contrast, or to add text and scale bars) was completed using Photoshop CS5 Extended. All measurements were made using the analytical features of Photoshop CS5 Extended.

High-resolution histological images of the cross-sections are digitally reposited online for scholarly use at MorphoBank (http://MorphoBank.org), project p494; see Table 2 for a list of slides and accession numbers. Digital images larger than 25,000 pixels in either dimension were digitally scaled (reduced to 17,000–20,000 pixels in the larger dimension) to allow processing on MorphoBank and because most image editing software does not support editing of gigapixel jpg files. These edits were made after scale bars had been added. Images in full resolution can be obtained from the author. All slides are reposited at the home institution of the specimens sampled (FMNH, MOR, OMNH).

**Table 2:** MorphoBank accession numbers of the high-resolution images for the slides used in this study.

Element	Specimen Number	Age	MorphoBank Accession Numbers
**Humerus**	OMNH 10144	J	M90594, M93560
	OMNH 2531	SA	M68276
	OMNH 8137	SA	M68277, M93561
	FMNH PR 2261	A	M68336, M93562
**Ulna**	OMNH 10144	J	M90596, M93563
	OMNH 2531	SA	M68279, M93564, M93565
	OMNH 34191	SA	M68280, M93566, M93567
	OMNH 62990	A	M68281, M93569
	FMNH PR 2261	A	M68278, M93568
**Femur**	FMNH PR 2263	J	M68273
	MOR 679	J	M68282, M93553
	OMNH 10144	J	M68283
	OMNH 34785	J	M68284, M93554
	MOR 682	SA	M68285
	OMNH 34784	SA	M75914, M93555
	FMNH PR 2261	A	M75796, M93556, M93692, M93693, M93694
**Tibia**	MOR 788	P	M68274, M93570
	OMNH 10144	J	M90615, M93571, M93572
	OMNH 34785	J	M68275, M93573
	OMNH 2926	SA	M68307
	OMNH 8137	SA	M75915
	OMNH 10134	SA	M68064
	OMNH 16563	SA	M75918
	OMNH 34783	SA	M75919
	OMNH 34784	SA	M68309, M68310, M68311, M93575, M93576
	OMNH 58340	SA	M75920
	OMNH 63525	SA	M68308, M93574
	OMNH PR 2261	A	M75921, M93577, M93578
**Fibula**	OMNH 34785	J	M90595, M93557
	OMNH 16563	SA	M68312, M93559
	OMNH 34783	SA	M68320, M93558
**Rib**	OMNH 16563	SA	M85890
	OMNH 63525	SA	M85891

For each specimen, the first number listed is a full cross-section, and any subsequent numbers are detailed images of one part of that closeup. Abbreviations: A adult, J juvenile, P perinate, SA subadult.

### Age class estimation

Apart from histological data, few reliable criteria for establishing ontogenetic stage using the external morphology of skeletal elements have been established for archosaurs [24], although various methods to describe the degree of ossification have been suggested as potential sources of ontogenetic information. Bone surface texture has been used to assess relative age classes (juvenile, subadult, adult) in living birds of known age [25], [26]. Based on these studies, bone surface texture has been used as a proxy for age class in ornithopods [25], nonavian theropods [25], [27], and ceratopsians [28], [29]. Dinosaurian age class assessments were often made using surface texture in combination with cranial suture fusion patterns, but have not yet been made in combination with skeletochronological (histological) assessments of age. Bone surface texture does not correlate with age or size in living *Alligator* [30], so this method is potentially problematic for fossil archosaurs in the absence of absolute age data. The correlation between age and the progression of neurocentral fusion has been established in living crocodylians [31], [32], with a similar sequence suggested for phytosaurs and aetosaurs [24]. However, neurocentral fusion sequences for dinosaurs are poorly understood [24], though ceratopsians [33], the sauropodomorph *Camarasaurus* [34], and living birds [35] are known to differ from living crocodylians. However, none of the nonavian dinosaur studies used specimens for which age had been estimated histologically, and most considerations of neurocentral fusion patterns have been based on one or two specimens, rather than an ontogenetic sequence [24].

In the absence of diagnostic criteria for ontogenetic staging, I initially selected specimens from the FMNH and OMNH collections based upon the relative sizes of the limb elements and features of the associated skeleton related to overall levels of ossification (e.g., bone surface texture, fusion of skull elements, neurocentral sutures, and/or ankle elements). Forster [11], [12] identified the individuals batch-catalogued as OMNH 10144 as a juvenile group (possibly arising from a single clutch of eggs) based on their extremely similar small size and their close association with each other and with an adult skeleton (OMNH 10132). Following her diagnosis, I initially termed OMNH 10144 and another specimen of similar size (OMNH 34785) as juveniles. OMNH 2531 also showed clear indications of skeletal immaturity (see discussion below), and I initially classified this as a large juvenile or small subadult. All other specimens were larger and harder to categorize based on skeletal morphology, so I sampled across the entire available range of sizes for the taxon.

Once the specimens were histologically sectioned, I grouped them into categories based on their tissue structure, following the histological age categories (perinate, juvenile, subadult, or adult) established in previous studies [16], [17], [36]. I distinguish between subadults and adults in the manner of Horner et al. [17], diagnosing as adults only those specimens that show histological evidence of skeletal maturity/senescence; that is, the presence of an external fundamental system (EFS) and/or extensive secondary remodeling of the mid- or outer cortex. In dinosaurs, skeletal maturity occurs well after the animal has reached reproductive maturity [37], [38]. I use “adult” to refer to skeletally mature specimens only, though some of my skeletally “subadult” specimens were likely reproductively mature [37], [38].

### Synonymy of Cloverly and Antlers specimens

Although the generic assignment of the Antlers specimens of *Tenontosaurus* is not contested, the specific referral of these materials requires some discussion. The synonymy of the Cloverly and Antlers *Tenontosaurus* specimens was questioned by Langston [7], based on his study of the skull of OU 8-0-S3 (since re-catalogued as OMNH 2531). He noted that this specimen had a “more camptosaurian profile, larger nares with a longer and more slender premaxillary bridge above the openings, and larger orbits” ([7]:p88), a lack of contact between the jugal and quadrate, and a secondary lateral fenestra bordered in part by the jugal rather than being entirely contained within the quadratojugal. As a result of these differences, Langston suggested that the Cloverly and Antlers specimens might not be conspecific. For this reason, Forster [11] did not include Antlers specimens in her redescription of *Tenontosaurus* postcrania. I reexamined the material Langston described, and do not think that the OMNH Antlers material can be excluded from *T. tilletti* using his criteria [7].

My reexamination of OMNH 2531 and other, well preserved skulls of *Tenontosaurus* collected from the Antlers Formation of Oklahoma indicates that OMNH 2531 is a large juvenile or small subadult. At approximately 20cm in length, the skull of OMNH 2531 is much smaller than the adult *Tenontosaurus* skulls from the Cloverly Formation (e.g., YPM 5456) and newly-discovered skulls from the Antlers Formation (e.g., OMNH 58340). Unfortunately, the skull of OMNH 2531 was not figured in Langston [7] in sufficient detail to confirm the contacts. Since Langston's assessment, that region of the skull of OMNH 2531 has been damaged, so the contacts of the jugal and quadrate can no longer be evaluated. However, the jugal and quadrate in all large subadult and adult skulls collected by the OMNH from the Antlers Formation contact each other as in the Cloverly specimens. The secondary lateral fenestra is entirely enclosed by the quadratojugal, and the relative proportions of the orbit and nares are also similar. The differences between OMNH 2531 and larger skulls are probably ontogenetic.

Langston ([7] also noted some differences in the ilium in the ornithopod material collected from the Antlers Formation of Texas (TMM 41508-1); the concavity of the dorsal margin and the angle of the anterior process were not as conspicuous as in the Cloverly *Tenontosaurus* specimens. He did not conclusively refer this individual to *Tenontosaurus*, though Winkler [4] subsequently referred TMM 41508-1 (transferred to DMNH and now recatalogued as DMNH 8386) to *Tenontosaurus* sp. My reexamination of this specimen suggests diagenetic alteration of the pelvis; for example, the dorsal margin of the ilium is not straight but bent laterally, and this alters the lateral profile of the element.

Adult and large subadult skulls from the Antlers Fm. (e.g., OMNH 16562, 58340) can be assigned to *Tenontosaurus* based on the following diagnostic combination of characters [8]: large external nares relative to orbit, subrectangular orbit, tall maxilla, slitlike antorbital fenestra, quadratojugal entirely containing second lateral fenestra, supraoccipital excluded from the foramen magnum, and downward-pointing tubers on the basisphenoid (personal observation). The Antlers *Tenontosaurus* cannot be assigned to *Tenontosaurus dossi*. All Antlers specimens for which skulls (OMNH 2531, 16562, 58340) and/or carpal elements (e.g., OMNH 2531, 16563, 58340) are available lack the autapomorphic premaxillary teeth and tightly interlocking carpus of *T. dossi* [8]. Some of the remaining diagnostic features of *T. dossi* (e.g., anterior flare of the scapula, scapula length relative to humerus) are ontogenetically variable in *T. tilletti* [11], whereas the others (e.g., convexity of the prepubic blade) are individually variable in *T. tilletti* [11].

Brinkman et al. [3] suggested that the Antlers form may represent a new species of *Tenontosaurus*, or an intermediate form between *T. tilletti* and *T. dossi*. However, they did not list any characters to support that conclusion. Given that the Antlers specimens can be assigned to *Tenontosaurus* using the published diagnoses [1], [8], [11] and that they cannot be assigned to *T. dossi* using the diagnosis of Winkler et al. [8], I treat the Antlers specimens as members of the type species (*T. tilletti*)—and therefore conspecific with the Cloverly specimens—in the absence of a thorough demonstration to the contrary. Future work may divide this taxon into more than one species, but because bone is an extremely conservative tissue, taxonomic revision should not affect the overall results of this study.

## Results

### Ontogenetic osteohistology of *Tenontosaurus*

I compared ontogenetic series of thin-sections from the mid-diaphysis of the humerus, ulna, femur, tibia, fibula, and ribs in order to assess the osteohistological differences through ontogeny for each element. In this section, I describe collagen orientation, vascular orientation/arrangement, osteonal development, and osteocyte orientation/arrangement separately, rather than using tissue-level descriptors such as ‘fibro-lamellar bone’ or ‘lamellar-zonal bone’. As originally intended, these terms each imply a suite of associated histological characters [39], but in recent years different authors have used different and inconsistent diagnostic criteria. These tissue-level descriptors are therefore somewhat limited in their comparative utility. The use of finer-level characters allows more specificity in comparative descriptions.

I described the osteohistology from direct observations of slides under the microscope. Histological terminology follows that of Francillon-Vieillot et al. [39], with the following clarification regarding how I made my diagnoses. Collagen orientation (lamellar, parallel-fibered, woven) was diagnosed using crossed Nicols and/or crossed plane polarizing filters. I inferred osteocyte orientation from the orientation of the long axes of the lacunae that housed them in life; I diagnosed lacunar orientation by focusing through the plane of the section under the microscope to determine the long axis of the cell. I diagnosed lines of arrested growth (LAGs) using two criteria: a break in bone deposition visible at multiple magnifications (determined by focusing through the hypothesized LAG) and the unbroken continuation of the line around the circumference of the bone section. In some cases, two or three lines running parallel to each other in extremely close succession are diagnosed as double LAGs or triple LAGs (respectively) because the space between them does not likely represent a full year of growth. The individual lines in these “LAG packets” share very subtle changes in morphology not shared by the previous or subsequent LAG, and are separated by more typical (larger) annual zones of deposition. Double and triple LAGs have been reported in other extinct and extant vertebrates [40]–[43], including other archosaurs [44], [45].

In addition to the standard terms of Francillon-Vieillot et al. [39], I use “vascular orientation” to describe how the long axes of vascular canals are oriented in the element (e.g., longitudinal, radial, circumferential, reticular). I use the term “vascular arrangement” to refer to the positions of the canals themselves relative to each other (e.g., “longitudinal canals arranged radially”). “Osteocyte orientation” refers to the position of the long axis of the bone cells relative to the long axis of the bone. “Osteocyte arrangement” refers to the position of the bone cells relative to each other and/or to vascular canals.

For all elements, measurements of cross-sectional and medullary cavity diameter were measured along major and minor axes, and cortical thicknesses were calculated by averaging the thickness along the major and minor axes of the bone. These measurements are presented in text and in Table 3. I discuss forelimb elements and then hindlimb elements. Within each element, specimens are discussed by age category in order of size. I start descriptions with overall bone dimensions, and then describe internal (endosteal/medullary) features, moving outward to the periosteal surface. For each element, at least one representative specimen is figured in full cross-section, with a detailed image of the primary cortical tissues. For other views (especially perimedullary and periosteal tissues), please see the supplemental images online at MorphoBank, project p494 (see Table 2 for accession numbers).

**Table 3:** Measurements of the cortex and medullary cavity for the cross-sections examined in this study.

Element	Specimen #	MinA	MajA	MCmin	MCmaj	ACT
**Humerus**	OMNH 10144	10.5	18.5	5	6.5	3
	OMNH 2531	28	?	14	?	7
	OMNH 8137	33.5	44	12	14	7
	FMNH PR2261	37.5	54	14	22	15.5
**Ulna**	OMNH 10144	7	10.5	2.5	4	2.5
	OMNH 2531	18	24	9	11	5.5
	OMNH 34191	22	36	5.5	7	9
	FMNH PR2261	26	45.5	8	16	13
	OMNH 62990	28.5	45	11	16	13.75
**Femur**	OMNH 10144	15.5	18	9.5	9.5	4
	MOR 679	21	21	11.5	16	4
	OMNH 34785	20	21	10	11.5	5
	FMNH PR2263	?	24	?	?	4
	MOR 682	?	47	?	26	10
	OMNH 34784	48.5	51	26	30	13
	FMNH PR2261	?	?	?	?	15–21+
**Tibia**	MOR 788	8	10	5.5	6	1
	OMNH 10144	13	15	5.5	6.5	4
	OMNH 34785	16	20	6	6.5	5.75
	OMNH 63525	34	40	15	17	10
	OMNH 10134	38	46	10	18	12.5
	OMNH 34784	39.5	46	11	12	15.25
	OMNH 2926	40.5	44	15	16	11.25
	OMNH 8137	43.5	45.5	14	19	13.25
	OMNH 16563	45	51	?	?	12+
	OMNH 34783	47	57.5	18	23	15.5
	OMNH 58340	44	55	19.5	22	13.25
	FMNH PR 2261	55	64	16	22	18.5
**Fibula**	OMNH 34785	7	9.5	3	3	2.25
	OMNH 16563	15.5	32	8	11	7
	OMNH 34783	24	31	7.5	9	10.25

For each element, all sectioned specimens are listed in size order (in the order discussed in the text). All measurements are in millimeters. Abbreviations: ACT average cortical thickness, MajA major axis diameter, MCmaj medullary cavity diameter along the major axis, MCmin medullary cavity diameter along the minor axis, MinA minor axis diameter.

### Humerus

Juvenile

Specimen examined: OMNH 10144

The collection of juveniles assigned to OMNH 10144 represents several individuals of the same size and likely from the same clutch [11], [12]. At least four individuals are present, based on the number of right femora. For each bone, all specimens measured within 3 mm of each other in total (proximal-distal) length, but only one of each of the major long bones was sampled to limit the amount of destructive sampling on rare juvenile material. Thus, the elements assigned to OMNH 10144 represent one or more individuals, but as they are sampled from individuals who were likely the same age at the time of death, they are treated as one individual for the purposes of this study.

**Figure 2:**
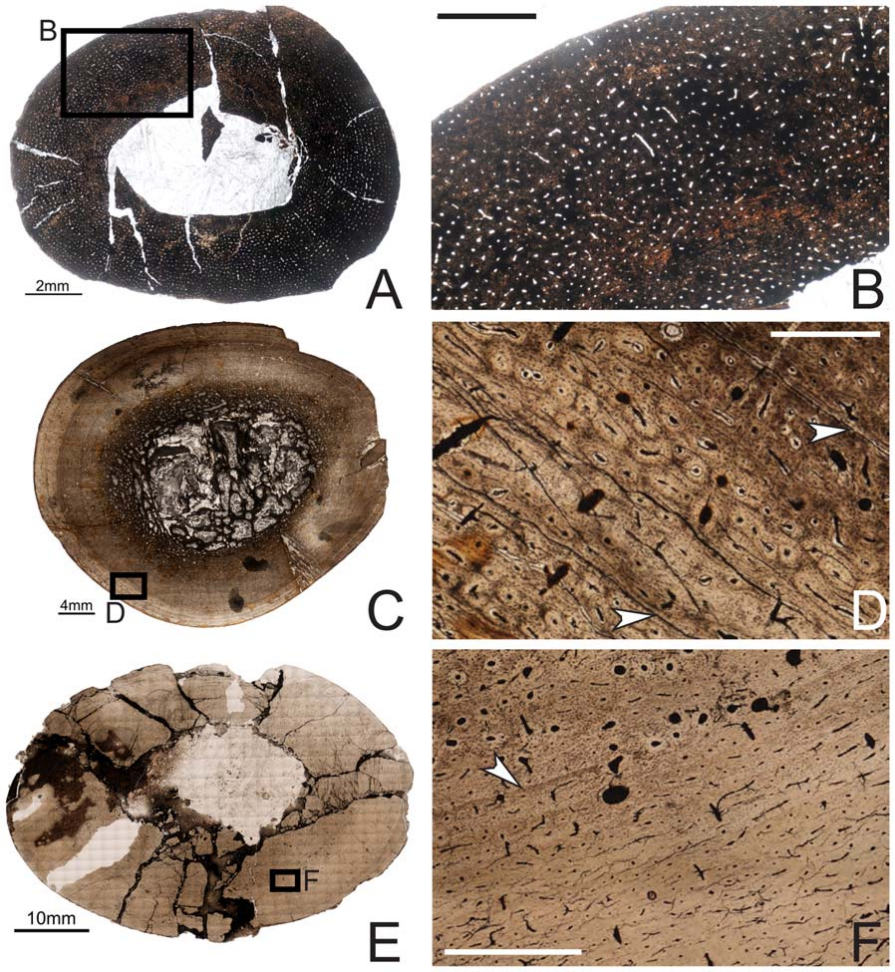
Osteohistology of the mid-diaphyseal humerus of *Tenontosaurus* in a juvenile (A, B), subadult (C, D) and adult (E, F). **A.** Cross-section of OMNH 10144. This bone was invaded by bacteria before fossilization and thus much of the primary tissue is obscured. It is presented here in cross-section to illustrate vascular density and arrangement. **B.** Detail of A, showing general vascular patterning. The cortex is dominated by longitudinal canals arranged circumferentially. **C.** Cross-section of OMNH 8137. **D.** Detail of C, showing primary cortical tissues. The bone is woven, and most canals are longitudinal primary osteons (some anastomose circumferentially). Two LAGs (arrows) are shown. **E.** Cross-section of FMNH PR2261. **F.** Detail of E, showing mostly primary tissues of the midcortex at a transition to slower growth. Deeper in the cortex (upper left), bone is woven and osteocytes are dense and disorganized. Some secondary osteons are visible, but they do not overlap or obscure all of the primary tissues. Past the LAG (arrow), canals remain dense but decrease in diameter, bone tissue is weakly woven, and osteocytes decrease in number and become more organized. Scale bars: A = 2 mm; B, F = 1 mm; C = 4 mm; D = 0.5 mm; E = 10 mm.

The mid-diaphyseal humerus of OMNH 10144 (Figure 2A, 2B) is elliptical in cross-section (midshaft diameter (MD): 10.5×18.5 mm, average cortical thickness (ACT): 3 mm), more strongly so than either the femur or tibia. The medullary cavity as preserved is also strongly elliptical (medullary cavity diameter (MCD): 5×6.5 mm). The histology of the cortex is largely obscured by bacterial invasion, preventing a complete description of the fabric of the bone or the osteocyte density/arrangement. The entire cortex of the humerus is extremely densely vascularized by longitudinally oriented simple canals of varying diameter and shape. These canals may have short radial and circumferential anastomoses. Overall, the canals are distributed in a more or less circumferential pattern. No LAGs, endosteal lamellae, or trabeculae are visible in this specimen.

Subadult

Specimens examined: OMNH 2531, 8137

OMNH 2531 is represented by a partial section comprising approximately three quarters of the total mid-diaphyseal circumference (see supplemental figures on MorphoBank). In cross-section it is shaped like a rounded triangle (MD: 28 mm, minor axis, ACT: 7 mm), whereas the medullary cavity as preserved is more square (MCD: 14 mm, minor axis) and lies centrally.

The medullary cavity of this humerus was filled by matrix, but during preparation this matrix was mechanically removed and the cavity subsequently filled with taxidermy epoxy (Kyle Davies, personal communication) to stabilize the element, which was broken in the midshaft region. As a result, the original endosteal surface is not preserved for most of this section. In one localized area, 3–7 endosteal lamellae are preserved. These cut into the cortical compacta and are crossed by three short radial canals.

No secondary remodeling is observed in this section. The entire cortex is comprised of woven bone tissue, well vascularized by primary canals. However, the tissue is more organized compared to the tissues of the subadult femur or tibia. Osteocyte density is consistently high throughout the cortex, and this density is similar in the interstices and around vascular canals. The dominant canal orientation in this element is longitudinal, and these canals are more or less arranged circumferentially. The vascular canals are all primary osteons with one or two cement lines, and these anastomose in about half the cases. Most anastomoses join two or three adjacent canals to form short circumferential canals, but longer radial anastomoses link five to nine canals throughout the cortex. Occasional oblique or reticulating canals are also present. Canal density does not seem to change throughout the cortex. Three LAGs punctuate this section (there are also three in this individual's ulna). All are quite distinct under regular and polarized light. The inner and middle LAG more or less divide the cortex into thirds, and the outermost LAG is very close to the periosteal surface.

The mid-diaphyseal humerus of OMNH 8137 (Figure 2C, 2D) also forms a rounded triangle in cross-section (MD: 33.5×44 mm, ACT: 7 mm), with a central, elliptical medullary cavity (MCD: 12×14 mm). A bony medulla lays endosteal to the compact bone of the cortex proper; this region is between 4–6 mm thick.

The medullary cavity is infilled by calcite crystals and clay matrix. The medulla is separated from the medullary cavity by several endosteal lamellae. The bony medulla was formed by the resorption of cortical tissues. There are several larger internal resorption rooms, but most, particularly in the periphery, are only three to four times the diameter of normal vascular canals. Little or no primary bone tissue is visible; secondary osteons comprise much of the visible bone. It is difficult to tell whether these areas were remodeled before or after the resorption began; most of the secondary osteons do not overlap each other, but on the other hand, almost none of the resorption rooms are lined with any lamellae.

The entire cortex is composed of woven bone tissues, but as in OMNH 2531, it is not as strongly woven as the bone tissues of the subadult tibia or femur. Osteocyte density is consistently high throughout the cortex, and while the osteocytes mostly are associated with vascular canals, not all are organized around them. The inner cortex shows a few secondary osteons, but the primary tissues are clearly visible, and secondary osteons do not extend into the mid- or outer cortical regions. Throughout the cortex, most of the canals are longitudinally oriented primary osteons, with some exhibiting short circumferential and occasionally oblique or radial anastomoses. These anastomoses are never long; rarely are more than three longitudinal canals connected to each other. The outer half of the outermost zone has almost no anastomoses in any direction. Four LAGs are visible in this specimen (compared to six in the tibia). All LAGs are obvious under both nonfiltered and polarized light, but they are thinner and less distinct compared to those of the tibia. Fewer LAGs may reflect growth differences between the humerus and tibia, but because the endosteal humerus is much more remodeled than the tibia, some LAGs may have been obscured.

Adult

Specimen examined: FMNH PR2261

The cortex of FMNH PR2261 is oval in cross section (Figure 2E, 2F; MD: 37.5×54 mm, ACT: 15.5 mm), while the medullary cavity as preserved is almond-shaped (MCD: 14×22 mm). In this specimen, there is no distinct medulla as in the subadult humerus, but the endosteal margin of the bone has been obliterated by clay matrix.

The inner cortex is extensively and heavily remodeled with secondary osteons; none of the original primary tissues remain. This remodeling extends into the mid-cortex, but no secondary osteons reach the outer third of the cortex. The primary bone tissues of the mid-cortex are composed of weakly woven to parallel-fibered bone, and the bone tissue of the outer cortex is almost exclusively lamellar. The canals mostly are longitudinally oriented primary osteons, with some very short circumferential, radial, or oblique anastomoses. These never connect more than two or three longitudinal canals. The mineral phase of the bone tissue immediately surrounding the canals is denser than the interstitial bone in the mid- and outer cortex. The diameter of the canals does not change periosteally, but there are fewer osteocytes in the mid- or outer cortex compared to the secondary osteons of the inner cortex or to any of the other elements. The histology of the outer cortex is primarily lamellar and interrupted by many LAGs, similar to that of the tibia and femur of FMNH PR2261 (NB: During photography, this slide, as well as slides of the tibia and femur of this specimen, was wet with oil to increase light penetration. The oil has a different refractive index than that of water and the water-clear resin in which the specimen was embedded. As a result, under regular transmitted light LAGs and other areas of higher mineral density are not as distinct as in these photos as they are in those of other specimens). In the outermost zones, a few thin bands of parallel-fibered can be found external to some LAGs, but woven bone is absent in this region. The zones between LAGs in the outermost cortex decrease in width periosteally, but all are extremely narrow.

Nine LAGs are visible in this section, but as in the tibia and femur, the extent of internal bone remodeling strongly suggests that several earlier/internal LAGs have been obscured. Some of the outermost zones are bordered by ambiguous LAGs that are more obvious under polarized light but less distinct under nonfiltered light.

### Ulna

Juvenile

Specimen examined: OMNH 10144

**Figure 3:**
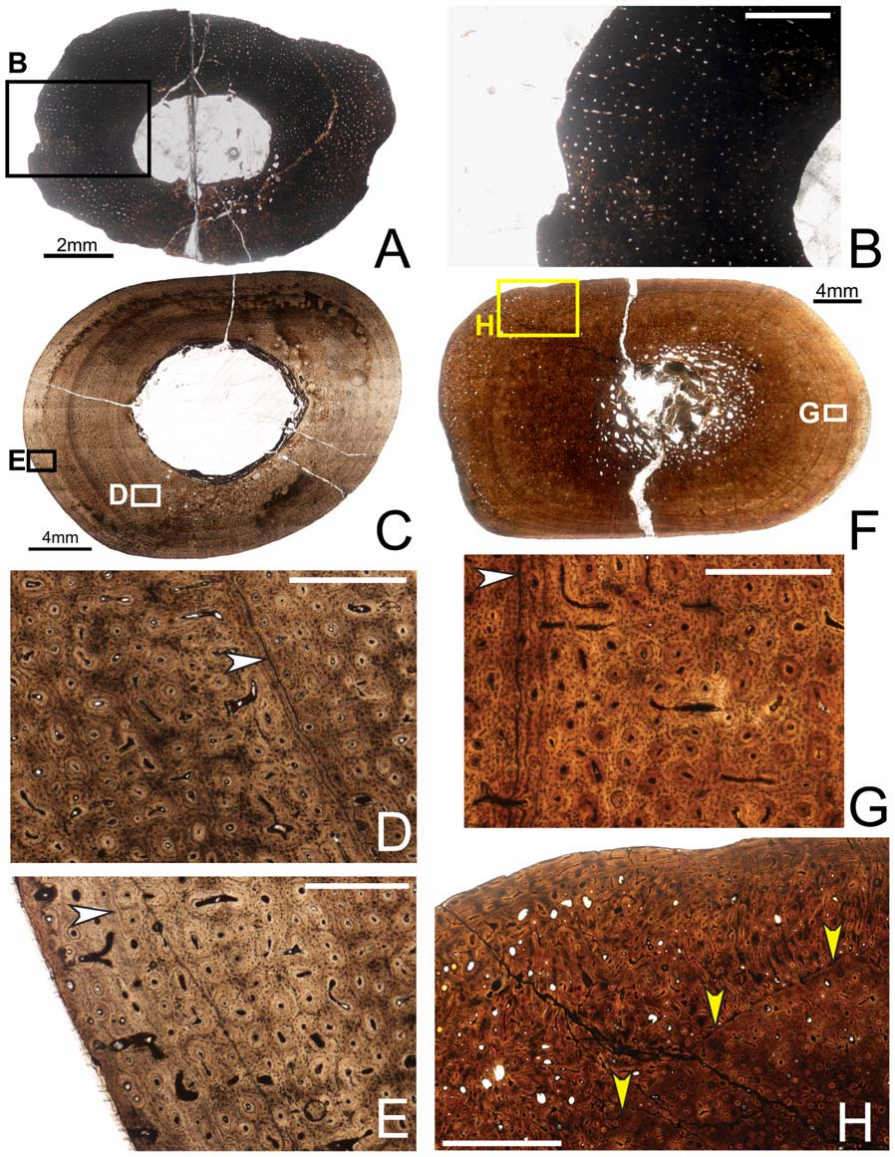
Osteohistology of the mid-diaphyseal ulna of *Tenontosaurus* in a juvenile (A, B) and two subadults (C–E, F–H). **A.** Cross-section of OMNH 10144. This bone was invaded by bacteria before fossilization and thus much of the primary tissue is obscured. It is presented here in cross-section to illustrate vascular density and arrangement. **B.** Detail of A, showing general vascular patterning. The cortex is dominated by longitudinal canals arranged radially. **C.** Cross-section of OMNH 2531. **D.** Detail of the midcortex of C, showing primary cortical tissues. Longitudinal primary osteons are visible in primary woven bone tissue. One LAG (arrow) is shown. **E.** Detail of the periosteal region of C, showing the surface condition in ulnae that lack the middiaphyseal rugosity. Longitudinal primary osteons are visible in primary woven bone tissue, very similar to the tissue of the midcortex. **F.** Cross-section of OMNH 34191. **G.** Detail of the midcortex of F, showing primary cortical tissues. Midcortical tissues are similar to those shown in OMNH 2531. One LAG (arrow) is shown. **H.** Detail of the periosteal region of F, showing the periosteal condition in ulnae with a rugosity on the posterior surface of the bone at midshaft. The histology of this rugosity is a strongly woven, high vascularized, and very disorganized tissue that builds along a surface similar to that seen in E (yellow arrows). It grades laterally into and is capped by more typical primary bone tissue identical to that of the rest of the cortex. Scale bars: A = 2 mm; B = 1 mm; C,F = 4 mm; D,E,G = 0.5 mm; H = 2 mm.

Like the humerus, the mid-diaphyseal ulna of OMNH 10144 (Figure 3A, 3B) is strongly elliptical in cross-section (MD: 7×10.5 mm, ACT: ∼2.5 mm), as is the medullary cavity (MCD: 2.5×4 mm), which sits centrally. This specimen suffers from levels of bacterial invasion similar to those seen in the humerus. The ulna is extremely densely vascularized, with longitudinally oriented simple canals arranged radially throughout almost the entire cortex. The canals anastomose radially in some areas (especially periosteally), but no true radial canals are present. Throughout most of the internal cortex, vascular canals are uniform in size and shape, but toward the periosteal surface of the cortex, some elongate circumferentially. As in the femur and tibia, a band of avascular lamellar tissue lines the endosteal surface of the cortex, and no trabeculae are visible. No LAGs are visible in this section.

Subadult

Specimens examined: OMNH 2531, OMNH 34191

OMNH 2531 and 34191 show differences in histology that may be related to size or morphological differences. Both individuals are from Antlers Formation localities. OMNH 2531 is the smaller individual and has a smooth shaft. OMNH 34191 is larger and has an oval rugosity (∼3×7 cm), with the long axis oriented down the long axis of the bone) on the posterior side of the midshaft. Although not reported by Forster [11], about half of the large (i.e., subadult or adult) *Tenontosaurus* individuals in the OMNH collection have a similar rugosity in the same location (personal observation). These include individuals from the Cloverly and Antlers formations, and the ulnae and associated elements show no other indications of skeletal pathology. Whether these rugosities represent a common individual variation, pathology, or age/sexual dimorphism, is not known.

OMNH 2531 is oval in cross-section (Figure 3C, 3D, 3E), with a large, centrally-place oval medullary cavity (MD: 18×24 mm, ACT: 5.5 mm, MCD: 9×11 mm). The medullary cavity was infilled by calcite crystals that have destroyed most of the original endosteal margin. About one fourth of this original margin remains, and it is lined with a very thick band of endosteal lamellae (20–25 lamellae thick).

Most of the primary cortical tissues are unremodeled, but secondary osteons and isolated resorption rooms occur in the deep cortex. Some of the resorption rooms are unlined by lamellae, but most have at least three lamellae. The primary cortical bone tissue consists entirely of woven bone in which the interstitial osteocytes are dense and disorganized. The vascular canals are all primary osteons, and most of these are longitudinal canals organized more or less circumferentially. About half of these canals anastomose with one, occasionally two, other canals, and these connect radially and circumferentially. The radial anastomoses are more common and more likely to link more longitudinal canals.

The cortex is punctuated by two or three LAGs. The innermost is a very distinct single LAG. Just external to this LAG, a very thin band of parallel-fibered bone was deposited before the woven bone that forms most of the zone. The middle LAG lies in an annulus of parallel-fibered bone tissue <0.1 mm thick. The outermost LAG is less distinct than the others under regular transmitted light, and very locally degrades into an annulus. External to this LAG, the vascular canals are a mix of primary osteons and simple vascular canals, and osteocytes are noticeably less dense, although they remain disorganized.

OMNH 34191 is a rounded rectangle in cross-section (Figure 3F, 3G, 3H). The cortex is very thick relative to the diameter of the bone (MD: 22×36 mm, ACT: 9 mm), and the medullary cavity very small (MCD: 5.5×7 mm). The medullary cavity was not invaded by crystals, but was infilled with a claystone matrix. This element exhibits an oval, bony medulla ∼3 mm thick, which sits off-center anteriorly. The medulla retains a distinct circular margin of 15–20 endosteal lamellae. The trabeculae in this region formed by secondary resorption of primary or heavily-remodeled cortical tissues and are substantial (0.1–.04 mm thick). The central core of these trabeculae may preserve some of the original cortical tissues, but all are lined with many (5–20) lamellae. The resorption rooms are also quite large (some as wide as 2–4 mm in diameter), but the margins are distinct. Though many resorption rooms are visible in this section, it does not appear that the element was undergoing significant resorption at the time of the animal's death.

The inner cortex of OMNH 34191 is heavily remodeled by secondary osteons. The remodeling is extremely dense in the posterior region of the bone, where many generations of secondary osteons overlap each other, but decreases in density anteriorly. In the anterior quadrant of the bone, many secondary osteons are visible, but primary cortical tissue can be viewed among them. The primary cortical tissues of the inner, mid-, and most of the outer cortex are very similar to those of OMNH 2531, consisting almost entirely of woven bone perforated by longitudinal primary osteons. One main difference between this element and OMNH 2531 is that the anastomoses are more developed; at least half the canals anastomose circumferentially or radially with one to four other canals. As in OMNH 2531, the radial segments tend to be longer than the circumferential ones. The other main difference is that the primary osteons are very distinct in OMNH 34191 (Figure 3G), with very sharp cement lines and one or two lamellae. The bone tissue of the outermost cortex is very similar to that of the inner and midcortex, except that the osteocytes are slightly less dense (though no more organized).

The posterior quadrant of OMNH 34191 shows a different histology external to the third LAG (Figure 3H). This tissue is highly disorganized woven bone tissue with no consistent pattern of vascular organization. The vascular canals are a mix of primary osteons and simple vascular canals oriented in all directions, and show a much larger diameter compared to more internal canals. Some longitudinal canals appear to be undergoing substantial resorption, but it cannot be determined whether the resorption is from heavy Haversian remodeling (i.e., the formation of new, large secondary osteons) or whether this region is becoming more cancellous. The osteocytes are extremely dense in this region; in places they are difficult to distinguish from adjacent bone cells. In places, the bone tissue is exceptionally dense in woven collagen fibers and shows a radial pattern of bone growth. The bone of this region appears at first glance to be pathological, but it is not laterally constrained or localized. The most “atypical” bone is positioned posteriorly and grades into normal bone on the medial and lateral sides of the element (i.e., towards the edges of the rugosity). The commonality of this rugosity among several individuals also makes a pathological diagnosis less certain.

Three LAGS punctuate the cortex of OMNH 34191. The innermost LAG locally forms a double LAG with the two portions spaced 0.2–0.4 mm apart. The midcortex LAG is a single LAG that locally becomes a tight packet of two to three LAGS 0.04 mm apart. The outer LAG is a double LAG through most of its circumference, and between the two lines (spaced 0.2–0.4 mm apart), the bone is parallel-fibered and shows lower levels of vascularity.

Adult

Specimens examined: FMNH PR2261, OMNH 62990

**Figure 4:**
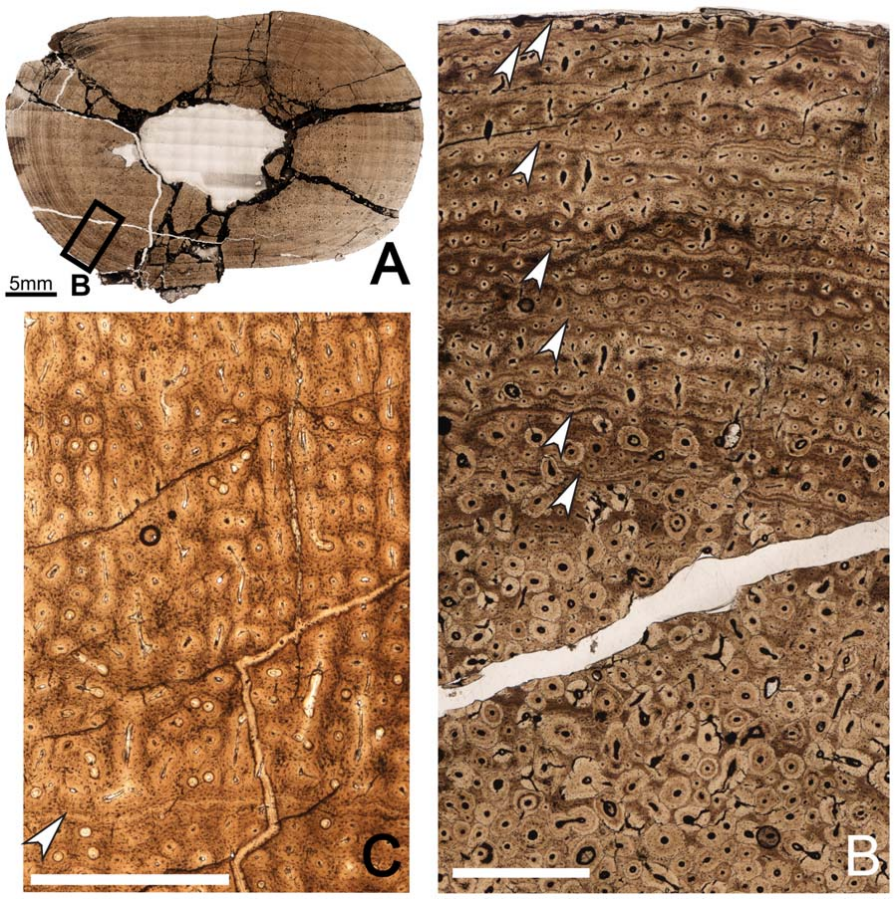
Osteohistology of the mid-diaphyseal ulna of *Tenontosaurus* in two adults. **A.** Cross-section of FMNH PR 2261. **B.** Detail of A, showing primary and secondary tissues of the mid- and outer cortex. The midcortex experiences dense secondary remodeling, and the outer cortex shows longitudinal primary osteons in parallel-fibered bone tissues grading into longitudinal simple canals in lamellar bone. Seven LAGs (arrows) are shown. **C.** Detail of OMNH 62990 (no cross-section shown), illustrating the radial arrangement of longitudinal primary osteons in an unremodeled area of the midcortex. One LAG (arrow) is shown. Periosteal surface to the top of this image. Scale bars: A = 4 mm; B,C = 1 mm.

The histology of FMNH PR2261 (Figure 4A, 4B) and OMNH 62990 (Figure 4C) are quite similar. The cortices and medullary cavities of both individuals are oval in cross-section (FMNH PR2261: MD: 26×45.5 mm, ACT: 13 mm, MCD: 8×16 mm; OMNH 62990: MD: 28.5×45 mm, ACT: 13.75 mm, MCD: 11×16 mm), and the medullary cavities sit at the geometric center. OMNH 62990 has been diagenetically “exploded” somewhat by the clay matrix that infills the medullary cavity.

Matrix infill has removed the endosteal margin in both individuals. In both ulnae, the inner cortex is densely remodeled with secondary osteons that have replaced all primary bone tissues; these osteons overlap each other throughout the region. The mid-cortex and outer cortex are very similar to each other histologically. These regions are composed of weakly woven bone and the vascular canals are almost exclusively longitudinally oriented primary osteons. The longitudinal canals are arranged in a strongly radial pattern through the entire cortex (Figure 4C), as in the juvenile. In both individuals, some of the radial rows anastomose along part or most of the row, forming radial canals. The radial canals are more or less evenly spaced, with two to four radial rows of longitudinal canals between them. The anastomoses are longer in OMNH 62290. Occasional circumferential anastomoses connect two adjacent longitudinal canals in places. As in the adult humerus, the bone immediately surrounding the canals is more densely mineralized than the interstitial bone. In the outermost cortex, vascularity decreases dramatically.

Seven LAGs occur in the cortex of FMNH PR 2261 and five to six in OMNH 62990. All LAGs are restricted to the outer cortex (secondary remodeling obscures the inner and midcortex). The LAGs are thin as in the other adult specimens and are usually associated with thin bands of densely mineralized avascular tissue separating zones of vascularized bone tissue. The zones between these annuli contain weakly-woven or parallel-fibered bone almost all the way to the edge of the element. In OMNH 62990, several lines occur in short succession, with only a single row of primary osteons separating them from the last LAG. In some areas, a thin band of poorly-vascularized, nonwoven bone occurs external to these lines. Regardless of whether these lines represent a true EFS or merely reflect extremely slow growth, it is likely that OMNH 62990 was at or very near to skeletal maturity.

### Femur

Juvenile

Specimens examined: FMNH PR2263; MOR 679; OMNH 10144, OMNH 34785

The femur of OMNH 10144 (see supplemental figures on MorphoBank) is more or less circular in cross-section (MD: 15.5×18 mm, ACT: ∼4 mm), and the medullary cavity as preserved is circular (MCD: 9.5×9.5 mm). As in the other elements of OMNH 10144, the medullary cavity is infilled with calcite crystals and bacterial invasion obscures much of the cortical tissues. A patch of cancellous bone sits among the crystals filling the medullary cavity of this specimen, but it is difficult to determine whether this bone lies in its original position, because it is not connected to the endosteal margin. The trabeculae are composed of primary woven bone tissues lined by zero to two endosteal lamellae, but the unlined interstices between trabeculae show no signs of resorption. The calcite crystals appear to have obliterated much of the original endosteal margin, but two to three endosteal lamellae are preserved in one small region. The femoral cortex of OMNH 10144 is densely vascularized by longitudinal canals. Where tissue is not invaded, all of the visible bone tissue is woven, and the vascular canals are all simple canals. The canals are circular in cross-section toward the center of the bone and become more elliptical toward the periosteal surface. The longitudinal canals are arranged in circumferential “rows” throughout the entire cortex, but in some areas a radial pattern can be observed as well. Vascular organization decreases periosteally. Endosteally, vascular canals are enlarged in diameter relative to those of the outer cortex, which are more or less uniform in size. No LAGs are visible in this specimen.

**Figure 5:**
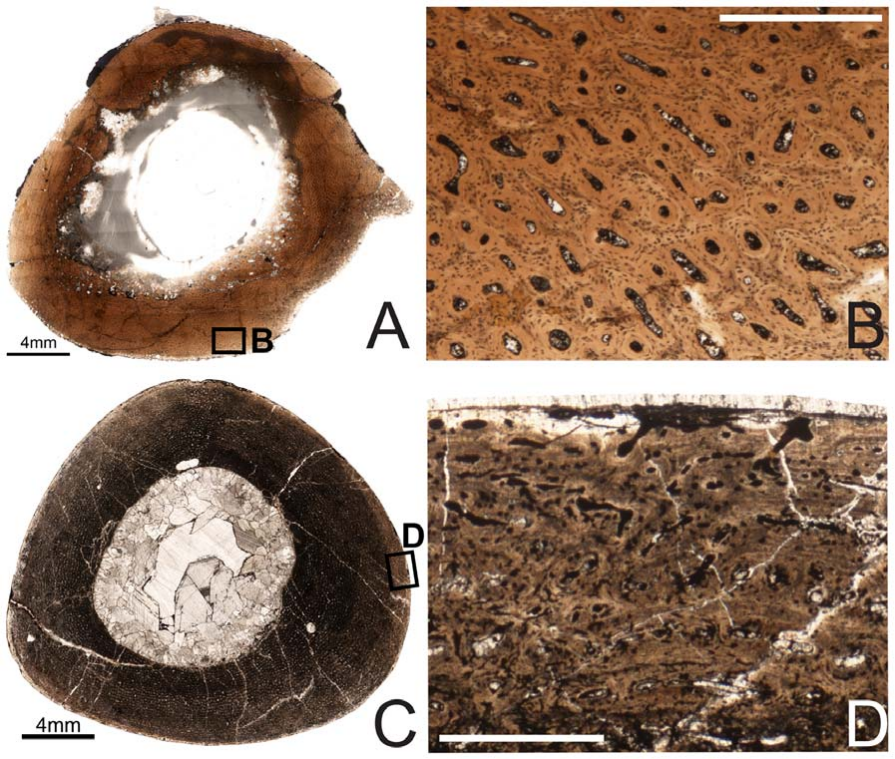
Osteohistology of the diaphyseal femur of *Tenontosaurus* in two juveniles. **A.** Cross-section of MOR 679. This section is slightly more proximal compared to others in this sample and shows part of the fourth trochanter. **B.** Detail of the outer cortex of A, showing primary cortical tissues. Longitudinal primary osteons in woven bone do not show many anastomoses. **C.** Cross-section of OMNH 34785 at the mid-diaphysis. **D.** Detail of the periosteal surface of C, showing longitudinal primary osteons and simple canals. Some canals open to the surface of the bone, but this is rare around the entire surface. Scale bars: A,C = 4 mm; B,D = 0.5 mm.

Horner et al. [17] described the osteohistology of one juvenile, MOR 679, which I re-examined for this study. MOR 679 comprises several elements from at least four individuals collected together, including several complete and partial elements. Although it was identified as a tibia in the Horner et al. [17] study, the fourth trochanter is visible in some thin sections and on the cast of the original element, and I re-identify it as a femur. Because the fourth trochanter is visible, this section is slightly more proximal than the rest of the femoral sections examined here. The femur is more or less circular in cross-section (Figure 5A, 5B; MD: 21×21 mm, ACT: 4 mm), with an oval medullary cavity (MCD: 11.5×16 mm). This femur also preserves trabecular structure in the medullary cavity. The trabeculae are composed of secondary/remodeled tissues. The interstices are all lined with multiple lamellae exhibiting distinct cement lines. The “cores” of the trabeculae preserve little primary bone tissue; rather, they are composed of many secondary osteons that overlap each other in some places. No lamellae separate the trabeculae from the cortex.

As Horner et al. [17] noted, the cortex of MOR 679 is entirely composed of woven bone tissue, osteocytes are dense throughout the cortex, and no LAGs are visible. The osteocytes are primarily organized around the vascular canals, but many occur in the interstices between vascular canals, and these lack any preferred organization. The vascular canals are mostly longitudinally oriented simple canals, but in some areas (e.g., the posterior quadrant near the trochanter), they anastomose radially. The canals are never surrounded by true lamellae with cement lines, although the bone tissues immediately adjacent to the canals are much less woven in texture. The vascular canals have a fairly uniform diameter throughout the cortex, except near the fourth trochanter, where the canals near the endosteal surface are about twice as large as those of the internal cortex. These larger canals sometimes open to the surface of the bone, but this only occurs on the trochanter and adjacent surface. Elsewhere the bone surface texture is more even.

The histological features of OMNH 34785 and FMNH PR2263 are very similar. The mid-diaphyseal femur of OMNH 34785 (Figure 5C, 5D) forms a rounded triangle in cross-section (MD: 20×21 mm, ACT: 5 mm), whereas the medullary cavity as preserved is slightly oval (MCD: 10×11.5 mm). The femur of FMNH PR2263 (see supplemental figures on MorphoBank) is incomplete in cross-section; only about three-fourths of the section is preserved (major axis diameter: 24 mm, ACT: 4 mm).

In FMNH PR2263, the bone tissue is composed entirely of primary woven bone tissue, and no LAGs are visible in the cortex. Most of the bone tissue in OMNH 34785 has been altered diagenetically by bacterial invasion, but some tissues are visible, especially toward the periphery. Only simple vascular canals are present periosteally, but endosteally, some primary osteons are present (this is harder to discern in OMNH 34785, given the extensive bacterial invasion). Throughout almost the entire cortex of both individuals, the vascular canals are primarily longitudinal in orientation, with few anastomoses. Although these anastomoses occur in all directions, they generally form radially in the internal cortex, and these are all very short. There are more radially anastomosing canals in the inner cortex of FMNH PR2263. In the outermost cortex, the anastomoses are longer and primarily form circumferentially. In OMNH 34785, some endosteal lamellae may line the medullary cavity, but these are difficult to discern. These features are not preserved in FMNH PR2263.

Subadult:

Specimens examined: MOR 682; OMNH 34784

MOR 682 is represented by a partial section preserving approximately half of the total circumference (see supplemental figures on MorphoBank). The mid-diaphyseal cortex of MOR 682 as preserved appears circular in cross-section (MD: 47 mm, major axis, ACT: 10 mm), with a large, circular medullary cavity (MCD: 26 mm, major axis) positioned centrally. This medullary cavity is infilled with calcite, but broken pieces of trabecular bone are visible between some of the crystals. Some of these pieces are similar to the embryonic/perinate tissues observed in the perinate tibia of MOR 788 in their shape and organization. The cores of the bone spicules in these trabecular pieces are composed of woven, primary bone tissues, occasionally pierced by simple canals. Unlike MOR 788, one or two lamellae line each of the cavities.

The calcite crystals obscure most of the endosteal margin of the cortex, and it is unclear whether any endosteal lamellae were present prior to crystal infiltration of the medullary cavity. The inner cortex shows a mix of longitudinally oriented simple canals and primary osteons. The canals do not show much organization and may anastomose radially or more often circumferentially with one or two adjacent canals. In one region, a few isolated secondary osteons are visible. These occur only in a region undergoing small amounts of medullary expansion by cortical resorption, as indicated by the presence of vascular canals with secondarily expanded diameters. The mid- and outer cortex is composed entirely of longitudinally oriented simple canals in an unremodeled woven bone matrix, although bacterial invasion obscures some of the finer histological details, especially in the mid-cortex. The vascular canals show a strong circumferential organization, with a secondary radial signal in some areas. These regions exhibit longer anastomoses compared to the inner cortex, and these are almost all radial connections of three or more longitudinal canals. In both the inner and mid-cortex, the diameter of the vascular canals is comparable in size to the large canals observed in all juvenile elements sampled in this study, but the canals are smaller in the outer cortex. Two LAGs are visible in this specimen; the outer LAG is a double LAG.

**Figure 6:**
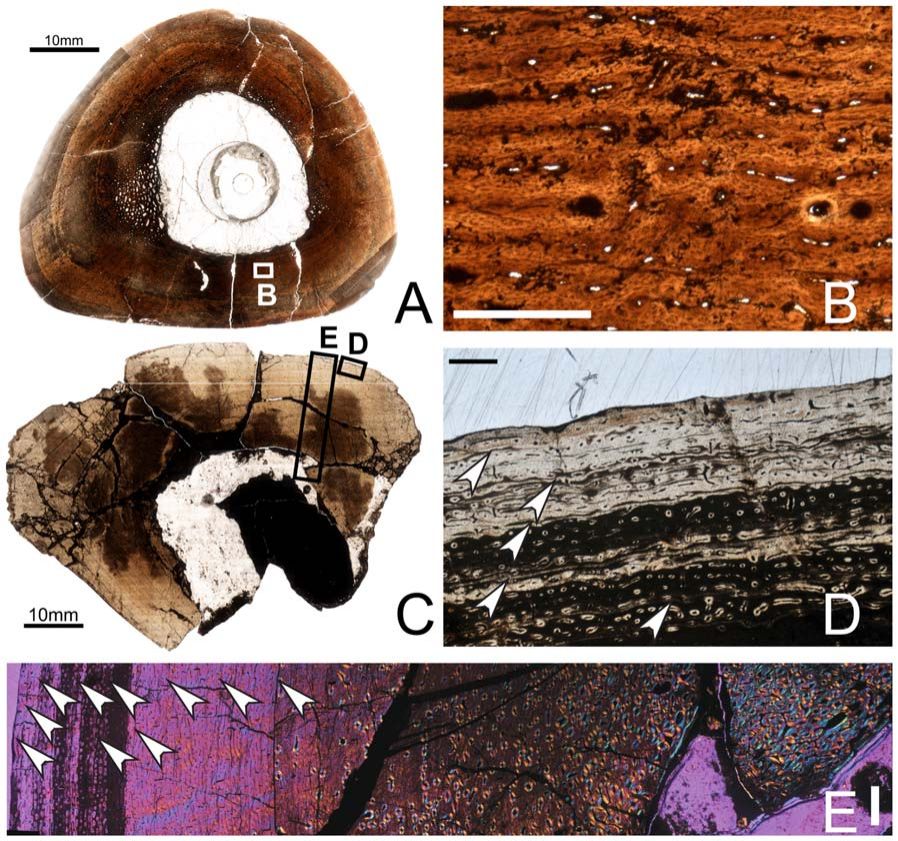
Osteohistology of the mid-diaphyseal femur of *Tenontosaurus* in a subadult (A,B) and adult (C–E). **A.** Cross-section of OMNH 34784. **B.** Detail of A, showing the primary cortical tissues of the cortex. Longitudinal primary osteons begin to form circumferential anastomoses in the woven bone tissues of the inner and midcortex. **C.** Partial cross-section of FMNH PR2261. **D.** Detail of C, showing the tissues of the periosteal region. Longitudinal primary osteons and simple canals are not as dense as in the midcortex and anastomose less frequently moving periosteally. Tissue is lamellar. Five LAGs (arrows) are shown. **E.** Detail of a radial transect through the cortex of C. Image taken through waveplate polarizing filters (crossed Nicols). Dense secondary remodeling is visible into the midcortex and zones of decreasing width are visible. Ten LAGs (arrows) are shown. Scale bars: A,C = 5 mm; B,D = 0.5 mm; E = 1 mm.

The cortex of OMNH 34784 is triangular in cross-section (Figure 6A, 6B; MD: 48.5×51 mm, ACT: 13 mm), with a relatively large, circular medullary cavity (MCD: 26×30 mm) positioned more or less at the geometric center of the bone. The histology of the femur is similar to that of the tibia of OMNH 34784, but there is some minor diagenetic alteration of the femur by bacterial invasion in the outer cortex (most of the cortex is not obscured, however). The cortex is composed of unremodeled/primary woven bone tissues rich in osteocytes. As in the tibia, active medullary expansion by means of resorption of primary cortical tissues is apparent, but endosteal lamellae separate these regions from the medullary cavity proper. Much of the medullary cavity is infilled with calcite crystals, but in a few areas a small amount of medullary bone tissue is preserved endosteal to these lamellae.

Unlike the tibia of OMNH 34784, a mix of primary osteons and simple canals occurs throughout the cortex, though simple canals are more common in the outermost cortex. Secondary osteons of varying size scattered throughout the internal cortex; these are densest closest to the endosteal margin. There are many longitudinally oriented canals throughout the cortex, but in the inner and mid-cortex, almost all anastomose circumferentially. As in the tibia, the longest of these circumferential canals connects perhaps six to seven longitudinal canals. In the mid-cortex, short radial anastomoses are also present. Secondary osteons of varying size are scattered throughout the internal cortex; these are densest closest to the endosteal margin. Very few secondary osteons can be found in the mid-cortex, and this occurs only in one region of the cross-section. In the outer cortex, most of the vascular canals are longitudinally oriented simple canals, with less circumferential anastomoses connecting them. There does not appear to be any change in osteocyte density through the cortex.

Fewer LAGs are present in the femur than in the tibia of the same individual (four in the femur compared to five to six in the tibia). This is consistent with observations in other ornithopod dinosaurs, who may record a different number of LAGs in different skeletal elements (see [16], [36] for discussion).

Adult:

Specimen examined: FMNH PR2261

FMNH PR2261 is represented by a partial cross-section (Figure 6C, 6D, 6E), which does not preserve the entire cortex spanning either the major or minor axis. The ACT in this region also cannot be determined, but is at least 15–21 mm. The medullary cavity is infilled with calcite crystals, and the original endosteal margin is not preserved. As with the slides of the humerus and tibia of this individual, this slide was wet with oil during photography in order to increase light penetration, and the difference in refractive index results in less distinct images of thin but mineralized areas (e.g., LAGs, cement lines) under regular transmitted light.

The primary bone of the deep cortex is obscured by several generations of secondary osteons (Figure 6E). This remodeling is not as visible in Figure 6C as it is in Figure 6E, but this is a result of the oil used during photography and the light filters used. A few resorption rooms are present in the deep cortex, but these are greatly outnumbered by the much smaller secondary osteons. The secondary osteons occasionally anastomose with each other, and these communications may occur in any direction.

The midcortex experiences some local remodeling, but it mostly consists of woven primary bone tissue perforated by primary osteons. In general, the midcortex shows a plexiform pattern of truly laminar bone (*sensu* [39]). There are many longitudinal canals, but nearly all anastomose circumferentially with several (three to ten) other longitudinal canals, and short radial anastomoses are also common. The radial canals tend to be of larger diameter compared to the circumferential canals. Osteocytes are very dense and very disorganized throughout the midcortex.

The outer cortex is less densely vascularized and shows lower osteocyte density compared to the midcortex. The reduced vascular density is achieved both by fewer anastomoses and by smaller diameters in both the longitudinal and circumferential primary osteons. The bone tissue in this region transitions from weakly-woven to parallel-fibered bone, and the outermost 0.2 mm (including the periosteal surface) is comprised entirely of lamellar bone (Figure 6D) with relatively few osteocytes.

The posterior quadrant of the bone is organized differently compared to the rest of the cortex. Here, the canals are much larger in diameter, primarily anastomose radially or in a reticulate pattern, and the primary tissues remain unremodeled. The bone was sampled at midshaft (distal to the fourth trochanter), but it is possible that either the M. caudofemoralis or the M. femorotibialis, which insert and originate (respectively) just proximal to this region, could have biomechanically influenced the histology.

Ten LAGs punctuate the cortex of FMNH PR2261. Four are widely but evenly spaced and sit in the mid-cortex, and six are more closely spaced and occur in the outer cortex. The LAGs are most easily visible under plane-polarized light because changes in mineral density darken the appearance of the cortex. No EFS is preserved in this specimen, but the narrower zones of the outer cortex combined with the lamellar bone tissue near the surface suggest that this animal was nearing the end of its phase of active skeletal growth [46]. Given the large number of secondary osteons in the inner cortex, as well as ontogenetic expansion of the medullary cavity, it is highly likely that more LAGs existed in this specimen but were obliterated by remodeling.

### Tibia

The tibia is the element most extensively sampled for this study. In *Tenontosaurus*, the mid-diaphyseal tibia undergoes the least amount of external morphological change, and there are no large muscle attachment sites at the mid-diaphysis.

Perinate

Horner et al. [17] described the osteohistology of the tibia of one perinate, MOR 788, which I re-examined for this study.

**Figure 7:**
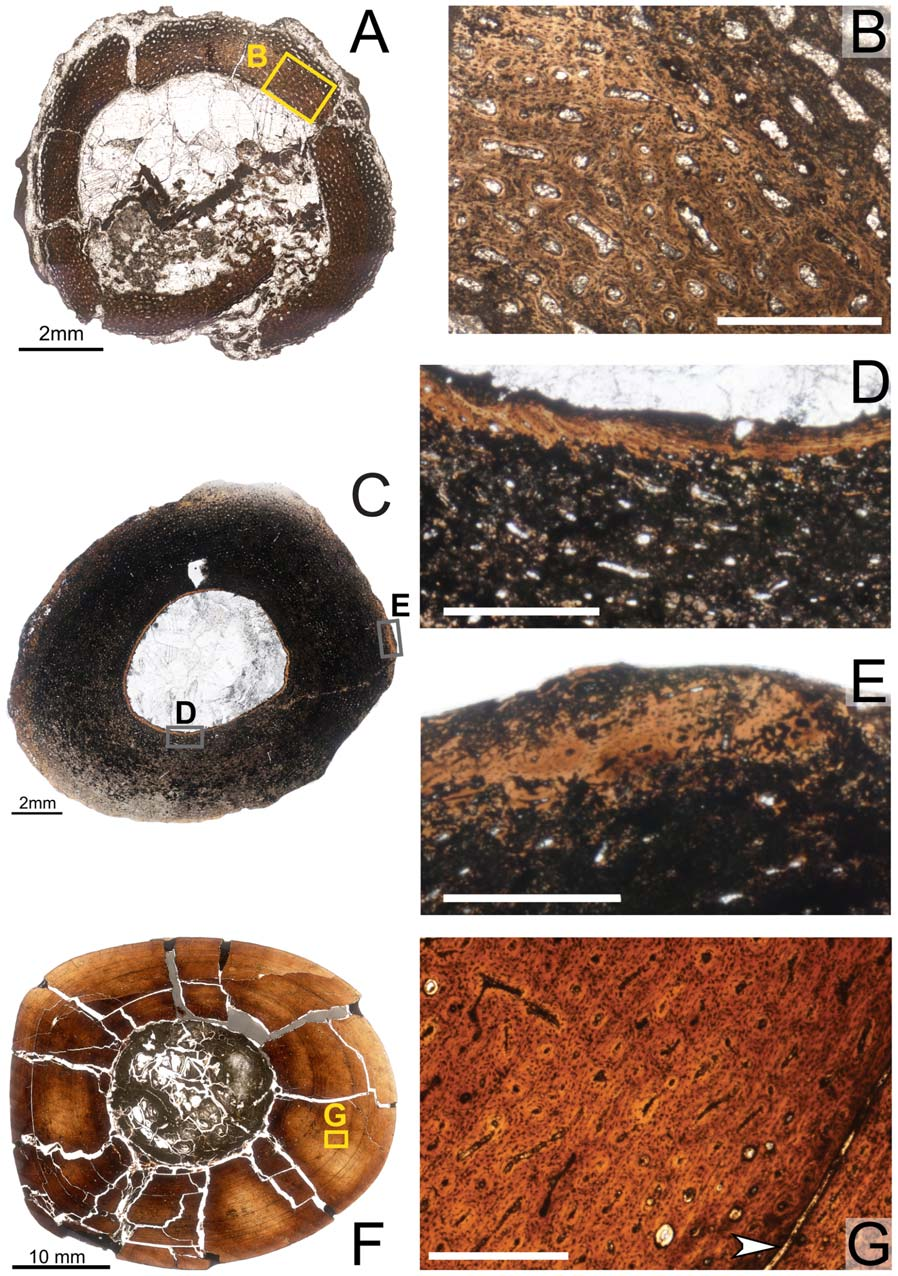
Osteohistology of the mid-diaphyseal tibia of *Tenontosaurus* in a perinate (A,B), juvenile (C,D), and subadult (F,G). **A.** Cross-section of MOR 788. **B.** Detail of the primary cortical tissues of A. Longitudinal simple canals and primary osteons have wide diameters compared to those of older ontogenetic age. Bone tissue is woven-fibered. **C.** Cross-section of OMNH 10144. This bone was invaded by bacteria before fossilization and thus much of the primary tissue is obscured. It is presented here in cross-section to illustrate vascular density and arrangement. **D.** Detail of the endosteal region of C, showing lamellar tissues (to right of image). Canals are narrower in diameter compared to the perinate (B). **E.** Detail of the periosteal region of C showing primary cortical tissues. The longitudinal primary osteons are surrounded by woven bone tissue. **F.** Cross-section of OMNH 63525. **G.** Detail of the midcortex of F. Longitudinal primary osteons run through woven bone tissue and show short circumferential anastomoses. One LAG (arrow) is shown. Scale bars: A,C = 2 mm; B,D,E,G = 0.5 mm; F = 5 mm.

The tibia of MOR 788 (Figure 7A, 7B; MD: 8×10 mm, ACT: ∼1 mm, MCD: 5.5×6 mm) is thin-walled and is largely infilled with calcite, but some trabeculae are preserved in the medullary cavity. Of these, most are fragmented, but some remain intact. The trabeculae strongly resemble the cortical bone tissues of embryonic archosaurs such as *Alligator*, *Maiasaura*, and *Orodromeus* [16], [47] in their shape and connectivity, and are likely remnants of the embryonic cortical tissue. The trabeculae have cores of primary tissue rich in osteocytes, and a single lamella lines each of the interstices. This is most obvious in the areas where trabeculae are preserved intact adjacent to the cortex, but also can be seen in some of the fragmented bits between calcite crystals. The cores of the trabeculae are composed of woven bone with dense, flattened, elongate osteocytes. Small simple canals are present at some of the nodes between trabeculae. No resorption lines are visible, suggesting that the lamellae were deposited along the edges of the original bone tissue at some point after the original tissues of the cores formed. No lamellae separate the trabeculae from the cortex. The transition between trabeculae and cortex likely represents a transition in the type of bone deposition pre- and post-hatching, and there is no evidence of a pause in bone deposition.

The bone histology of the cortex is similar to that of other perinatal archosaurs [16], [17], [47]. Although it is rich in vascular canals, the cortex of MOR 788 has much lower porosity compared to embryonic individuals of other archosaur taxa. It is composed entirely of woven bone dense in osteocytes. Horner et al. [17] noted that the osteocytes were concentrated and organized around vascular canals in the inner two-thirds of the cortex, and distributed more randomly periosteally. However, much of the outermost cortex in this specimen is obscured by post-mortem bacterial invasion. In areas where the tissues are less altered, the osteocytes are associated with vascular canals as in the internal cortex. As noted by Horner and his colleagues [17], no lines of arrested growth (LAGs) are visible in this individual.

The vascular canals in the cortex of MOR 788 are all either longitudinally oriented primary osteons or simple canals. The vascular canals of the cortex are deposited more or less circumferentially throughout, but there are local deviations from this pattern. The canals vary widely in diameter and shape (i.e., how circular, elliptical, or irregular they seem to be) throughout the cortex, but the canals of the mid-cortex are noticeably smaller in diameter than those of the internal or outer cortex. The overall vascular density in this region does not appear to decrease, however. This change in vascular patterning may reflect seasonal changes in growth or nutrient availability, but in the absence of other elements/individuals of this growth stage, I cannot eliminate the possibility of individual variation. As Horner et al. [17] noted, the vascular canals of the outermost cortex open to the surface of the bone, resulting in an immature/irregular bone surface texture.

Juvenile

Specimens examined: OMNH 10144, 34785

The tibia of OMNH 10144 is circular to slightly oval in cross-section (Figure 7C, 7D, 7E; diameter: 13×15 mm, ACT: 4 mm), and the medullary cavity as preserved is more circular in shape (MCD: 5.5×6.5 mm). A thin band of avascular, lamellar bone lines the endosteal margin of the cortex (Figure 7D), and the medullary cavity is entirely infilled with calcite crystals. No trabeculae are preserved. There is a large amount of bacterial invasion in this element, especially in the inner cortex. Where visible, the bone tissue of the cortex is always woven bone and dense in osteocytes, and no LAGs are apparent. The vascular canals are primarily simple canals, and are almost all longitudinally oriented, but some primary osteons occur along the endosteal margin (Figure 7E). The vascular canals vary in diameter and are distributed throughout the cortex with no regular pattern of density or arrangement; locally, the canals may be arranged irregularly, circumferentially, or radially. In some areas, small radial anastomoses are present. These canals never open to the outer bone surface as in the perinate tibia.

The mid-diaphyseal tibia of OMNH 34785 (see supplemental figures on MorphoBank) is oval in cross-section (MD: 16×20 mm, ACT: 5.75 mm), as is the medullary cavity (MCD: 6×6.5 mm), which sits slightly off-center. As with OMNH 10144, the histology of this tibia is largely obscured by post-mortem bacterial invasion, but the patterns of vascularity are clearly discerned. Where visible, the cortex is composed of woven bone tissue. Endosteally, the vascular canals are longitudinally oriented but often have short circumferential anastomoses. Periosteally, the canals are predominantly longitudinal without anastomoses, but locally there are areas of strong radial connectivity. The canals near the periosteal surface are more oval and often anastomose circumferentially. The osteocytes are arranged around the vascular canals, but very few canals exhibit any clear cement lines. No LAGs are visible in the cortex, and a thin band of two to three endosteal lamellae lines the medullary cavity. Large resorption chambers are visible in the inner cortex (internal to the lamellae), but these are localized to one region of the bone. These chambers do not appear to be finished with lamellae, so it is likely that bone was actively being resorbed in these regions when the animal died. It also appears that the shape/position of the medullary cavity was beginning to shift even at this young age.

Subadult

Specimens examined: OMNH 2926, 8137, 16563, 10134, 34783, 34784, 58340, 63525

The cortex of OMNH 63525 forms a rounded triangle in cross-section (Figure 7F, 7G; MD: 34×40 mm, ACT: 10 mm), whereas the medullary cavity is oval in shape (MCD: 15×17 mm) and sits more or less in the geometric center of the bone. The cortex is composed entirely of woven bone, and almost all of it is primary bone tissue, except for a few isolated secondary osteons in the endosteal region. However, most of the endosteal region of the cortex is primary bone tissue showing primary vascular canals. Most of the vascular canals of the middle and outer cortex are simple canals, but perhaps 20% of them are primary osteons lined with one to two thin lamellae and weak cement lines. Osteocytes are dense and organized around the vascular canals. Throughout the cortex, the dominant canal orientation is longitudinal, but many of the canals anastomose circumferentially with one to two adjacent canals. These circumferential anastomoses are primary canals. Isolated radial canals extend for approximately one-quarter of the radius of the cortex, but there are no long circumferential canals. Five LAGs are visible; two of these are double LAGs and one LAG is very close to the periosteal surface. The zones between these LAGs vary in thickness. The medullary cavity is infilled with matrix and the cortical-medullary transition is not preserved.

The tibia of OMNH 10134 (see supplemental figures on MorphoBank) was distorted postdepositionally, giving the bone a more triangular shape in cross-section (MD: 38×46 mm, ACT: 12.5 mm). The medullary cavity is oval in shape (MCD: 10×18 mm). Some trabeculae are preserved intact despite calcite filling of much of the medullary cavity; these appear to be thin spicules of avascular bone with several lamellae lining the interstices. Because the trabecular “cores” are so thin, it is difficult to assess whether these formed de novo, or whether they formed by resorption of primary cortical tissues. No endosteal lamellae line the medullary cavity or separate the trabeculae from the cortex.

The cortex is composed almost entirely of woven primary bone tissue, with little remodeling of the inner cortex. As in the smaller OMNH 63525, the innermost cortex is mostly composed of primary longitudinal canals with a few secondary osteons scattered throughout. Minor amounts of bacterial invasion obscure some regions of the innermost cortex, but the density and size of the secondary osteons appear comparable to OMNH 63525. Most of the middle cortex is composed of longitudinally-oriented primary osteons connected by long, circumferentially-oriented simple canals and primary osteons. Because many of the circumferential canals are histologically younger than the longitudinal canals they link, the circumferential canals may have formed secondarily as anastomoses between longitudinal canals rather than forming simultaneously with the circumferential canals. Regionally, small areas of radial canals also occur. Much of the outer cortex is similar to the middle cortex, but two to three rows of longitudinally oriented simple canals occur close to the periosteal surface. Four LAGs are visible in this individual; only the outermost is a double LAG. The penultimate LAG locally grades into an annulus of parallel-fibered bone, but it is continuous around the entire section and easily identified under regular and polarized light. Given the larger size and increased vascular connectivity observed in OMNH 10134, it could be older than OMNH 63525, despite preserving one fewer LAG.

The bone histology of OMNH 34784 was described briefly by Lee and Werning [37] because it exhibits medullary bone, a specialized bone tissue deposited in the marrow cavity of birds and other dinosaurs prior to egg-laying, and used as a labile source of calcium for eggshell production. Because it contains these reproductive tissues, OMNH 34784 must have been a reproductively-mature female. However, the bone histology and size of the animal indicate that this individual was still in an active phase of growth when it reached reproductive maturity [37].

**Figure 8:**
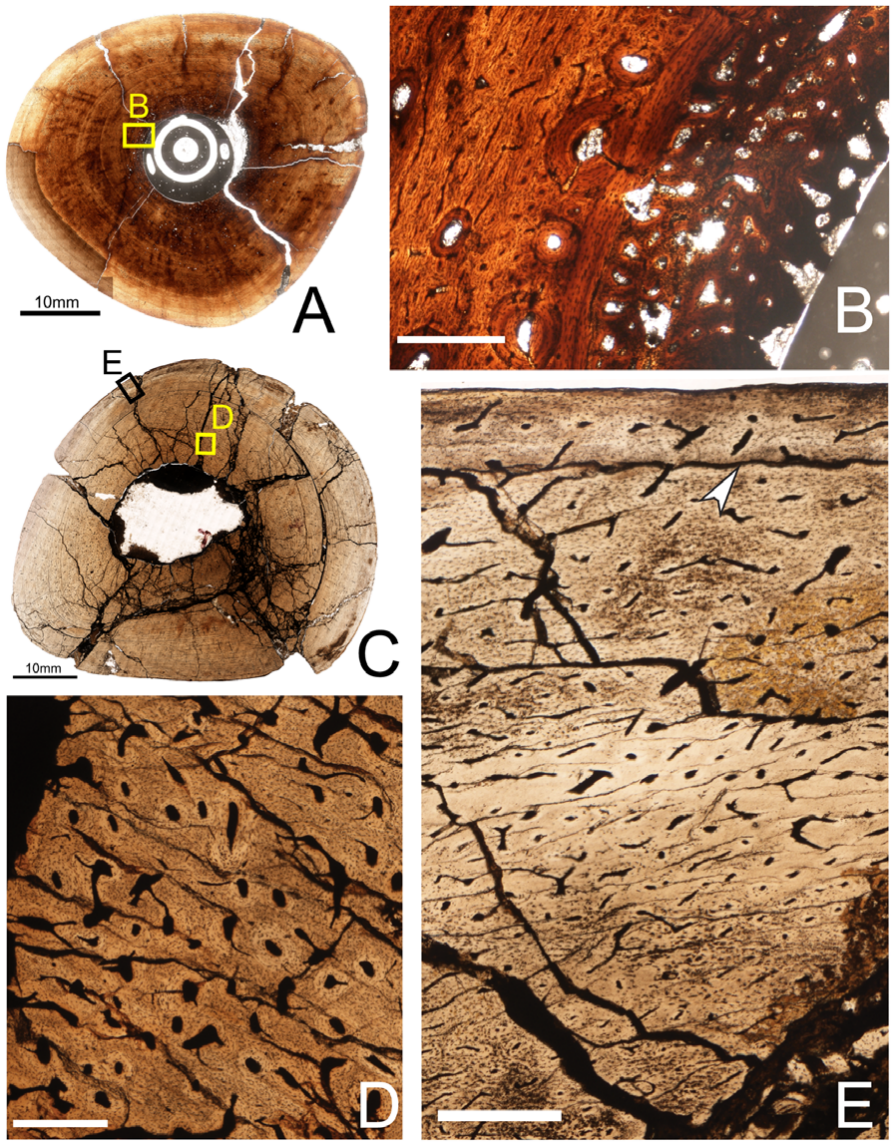
Osteohistology of the mid-diaphyseal tibia of *Tenontosaurus* in a subadult (A,B) and an adult (C,D,E). **A.** Cross-section of OMNH 34784. **B.** Detail of the inner cortex of A showing the endosteal surface and medullary bone (egg-laying) tissue. The primary cortical tissue (left side) consists of woven bone vascularized by longitudinal primary osteons connected by moderately long circumferential anastomoses. This tissue is beginning to undergo secondary remodeling. Endosteal lamellae separate the cortical bone from the medullary bone tissue, which radiates inward into the medullary cavity. **C.** Cross-section of FMNH PR 2261. This specimen was treated with oil before photography to increase light penetration, but this reduces the appearance of some thin, mineralized structures (LAGs, cement lines). **D.** Detail of C, showing histology of the inner cortex. Secondary osteons are abundant and obscure much of the primary cortical tissue. **E.** Detail of C, showing the outer cortex. Osteocytes are dense throughout the cortex, despite the transition to parallel-fibered bone in this region. Canals of the outermost cortex anastomose less frequently compared to the inner cortex. Scale bars: A = 5 mm; B,D,E = 0.5 mm; C = 10 mm.

The cortex of OMNH 34784 forms a rounded triangle in cross-section (Figure 8A, 8B; MD: 39.5×46 mm, ACT: 15.25 mm) and likely had an oval-shaped medullary cavity (MCD: ∼11×12 mm). Some of the medullary cavity was obliterated during macropreparation. A hollow metal rod was inserted into the medullary cavity and held in place by taxidermy epoxy in order to strengthen the bone across a break. The epoxy is visible in Figure 8A as the dark substance with surrounding two white circles at the center of the bone. The outer white circle is the position occupied by the metal rod. Despite this unfortunate damage to the medullary cavity surface enough of the original margin remains that its approximate size and general shape can be determined. Several endosteal lamellae separate the cortex from the medullary bone. This tissue is composed of radially-oriented spicules of primary bone tissue that extend into the medullary cavity. They were deposited after the formation of the endosteal lamellae and were not formed by resorption of primary cortical tissues. No external or histological indicators of disease are evident in this individual, and this tissue is also found in the femur of the same individual, so it is not likely pathological [37].

The cortex is composed of woven bone tissues rich in osteocytes, but the osteocyte density is slightly lower between the last LAG and the periosteal surface, compared to the rest of the cortex. The primary bone tissue of the inner cortex shows a mix of longitudinally oriented primary osteons, circumferential canals, and short radial and/or reticular canals. As in smaller individuals, these circumferential canals are common but are never extremely long; the longest connect perhaps six or seven longitudinal canals. Secondary osteons of varying size are scattered throughout the internal cortex; these obscure more of the primary tissues than in smaller individuals, but rarely obscure it all. In the mid-cortex, the vascular patterning changes in different areas; in some places, longitudinal canals dominate and in others, circumferential canals are much more common. Very few secondary osteons can be found in the mid-cortex, and this occurs only in one region of the cross-section. In the outer cortex, most of the vascular canals are longitudinally oriented simple canals, with less circumferential anastomoses connecting them.

Five LAGs are visible in OMNH 34784, and two are double LAGs. One of the double LAGs is additionally associated with an annulus. The woven texture of the bone changes through the zones separating them; the bone fabric is less woven in a thin band immediately following a LAG, but progressively becomes more woven approaching the next LAG. This indicates that *Tenontosaurus* did not grow at the same rate throughout the entire year, even during times of relatively rapid growth. This hypothesis is supported by the slightly lower osteocyte density in the outermost cortex.

The cortex and medullary cavity of OMNH 2926 (see supplemental figures on MorphoBank) are shaped similarly to those of OMNH 34784 (MD: 40.5×44 mm, ACT: 11.25 mm), but the medullary cavity as preserved is relatively larger (MCD: 15×16 mm). This is likely diagenetic; the clay-based matrix infilling the medullary cavity has destroyed some of the internalmost cortex. There is no evidence of the endosteal margin, and some crushing is apparent in some areas. Additionally, bacterial invasion obscures much of the internal cortex, and regionally, parts of the mid- and outer cortex as well. The bone that is preserved shows longer circumferentially-oriented primary osteons throughout the mid-cortex. The length of the circumferential canals decreases periosteally, and many longitudinally oriented canals occur in the outermost zone. As in OMNH 34784, osteocyte density decreases in the outer cortex, but these areas are still rich in osteocytes. Five to six LAGs are preserved in this specimen; at least one is a double LAGs. The zones between the last three are similar in width, but it is unclear whether or not the outermost LAG is a double LAG or represents two LAGs of narrower zonal width.

The cortex and medullary cavity of OMNH 8137, 16563, and 34783 (see supplemental figures on MorphoBank) are shaped similarly in cross-section to those of OMNH 2926 and 34784, although 16563 has been somewhat “exploded” diagenetically (8137: MD: 43.5×45.5 mm, ACT: 13.25 mm, MCD: 14×19 mm; 16563: MD: ∼45×51 mm, ACT: >12 mm, MCD: not determinable; 34783: MD: 47×57.5 mm, ACT: 15.5 mm, MCD: 18×23 mm). As for several elements of FMNH PR 2261, oil was used during photography of the tibia of OMNH 34783, which makes some features less distinct (e.g., LAGs, cement lines) under regular transmitted light.

The histology of the tibiae of these three individuals is very similar, so I discuss them together. In all individuals, the bone tissue is woven throughout the cortex; however, it is more weakly woven in the outermost zones (especially in OMNH 16563 and 34786). Much of the primary tissue of the inner cortex has been remodeled by secondary osteons, and secondary osteons are found scattered (never densely) into the mid-cortex. The primary tissue of the mid-cortex is dominated by circumferentially-oriented vascular canals, forming true laminae. The mid-cortex is dense in osteocytes; these are tightly associated with the vascular canals. OMNH 34783 shows a pattern similar to that seen in OMNH 34784; within each zone, the area where the bone is most woven occurs just outside the LAG, and depositional rate (as indicated by the woven texture of the bone) decreases through the year leading up to the next LAG. In all three individuals, the circumferential canals of the outer cortex are much shorter than those of the mid-cortex, and many more longitudinal canals can be found. In OMNH 16563 and 34783, the outermost zone is much thinner than the previous three or four zones, which are all approximately the same width.

OMNH 8137 preserves six LAGs, two of which are double LAGs; OMNH 16563 preserves eight to nine LAGs, one of which is a double LAG; and OMNH 34783 preserves at least six to nine LAGs, several of which may be double LAGs (making it difficult to assess LAG count). In all cases, the combination of secondary remodeling of the internal cortex and expansion of the medullary cavity has likely obscured or obliterated one or more LAGs, so the number of LAGs should not be taken as literal ages for these individuals. No trabeculae are preserved in either specimen, but the internalmost cortex of OMNH 8137 was undergoing medullary cavity expansion at the time of death. In one place a small strip of three endosteal lamellae is preserved, but the tissues internal to them are being resorbed. Large resorption rooms are visible; these are not lined by lamellae and the bone between them is consists of remodeled cortical tissues.

The cortex of OMNH 58340 (see supplemental figures on MorphoBank) is more rounded in cross-section but is not a true oval (MD: 44×55 mm, ACT: 13.25 mm), whereas the medullary cavity retains its oval shape (MCD: 19.5×22 mm). This is likely the result of diagenetic deformation, as the larger FMNH PR2261 retains the “rounded triangle” shape in cross section. OMNH 58340 may represent a late subadult or possibly an early adult. The primary cortical histology of the inner cortex is fairly similar compared to the other subadults, but it shows definite signs of slowing growth moving periosteally: the secondary osteons are more common in the mid-cortex, and even extend to the outer cortex in some areas. Additionally, the bone of the outermost cortex is less woven than that of the inner and midcortex; the bone transitions to weakly-woven and ultimately parallel-fibered bone through the mid- and outer cortex, and finally to lamellar bone.

Eight to nine LAGs are visible in the cortex, but the inner cortex was undergoing expansion at the time of death and LAGs were likely obliterated in the process. The LAGs of the midcortex all lie in annuli of parallel-fibered bone tissue and are fairly tightly spaced, with zones of very regular width between them. The LAGs of the outer cortex decrease in width periosteally. However, the more extensively remodeled inner cortex suggests a more mature individual compared to the other subadults. Though this individual is certainly closer to skeletal maturity than the other subadults, the histology is not as mature as that of FMNH PR 2261, and there is no EFS. I follow the methodology of Horner et al. [17] and Woodward et al. [46] in designating this individual as a subadult in the absence of that conclusive histological sign of skeletal maturity.

Adult

Specimens examined: FMNH PR2261

The cortex of PR2261 forms a rounded triangle in cross-section (Figure 8C, 8D, 8E; MD: 55×64 mm, ACT: 18.5 mm), with a small oval medullary cavity (MCD: 16×22 mm) that sits slightly off the geometric center of the bone. As in the humerus and femur of this individual, oil was used during photography, making some features less distinct under regular transmitted light.

Almost all of the innermost edge of the cortex has been obliterated by matrix infill, but one small area with one to two endosteal lamellae remains intact. The inner cortex is extensively remodeled with secondary osteons; very little of the original primary tissues remains visible. This remodeling extends well into the mid-cortex, and scattered secondary osteons reach even the outer third of the cortex. The primary bone tissues of the mid-cortex reveal woven bone with circumferential and short radial canals (forming short reticular anastomoses in some areas). As in younger individuals, the bone is more strongly woven immediately after a LAG, and becomes slightly less woven leading to the next LAG. In the outer cortex, the anastomoses are shorter, linking only two, three, or four longitudinal canals. These canals have a smaller diameter compared to the more internal canals. Here the fabric of the bone tissue also changes; the outer zones are composed of alternating bands of woven and parallel-fibered bone tissue. These transition in the outermost zones to a pattern of weakly woven or parallel fibered bone immediately after a LAG followed by parallel-fibered or nonwoven bone tissue. The nonwoven portions are more densely mineralized than the more internal portion; the histology here is extremely similar to the lamellar bone of subadult and adult crocodylians and other slow-growing pseudosuchians archosaurs (see [48], [49] for examples) and some dinosaurs (e.g., [50]). The zones between the LAGs of the outermost cortex decrease only slightly in width periosteally, but all are extremely narrow. At least eight LAGs occur in the outer cortex alone, but the great extent of internal bone remodeling and some distortion of the specimen makes it difficult to trace LAGs completely around the section.

### Fibula

Juvenile

Specimen examined: OMNH 34785

**Figure 9:**
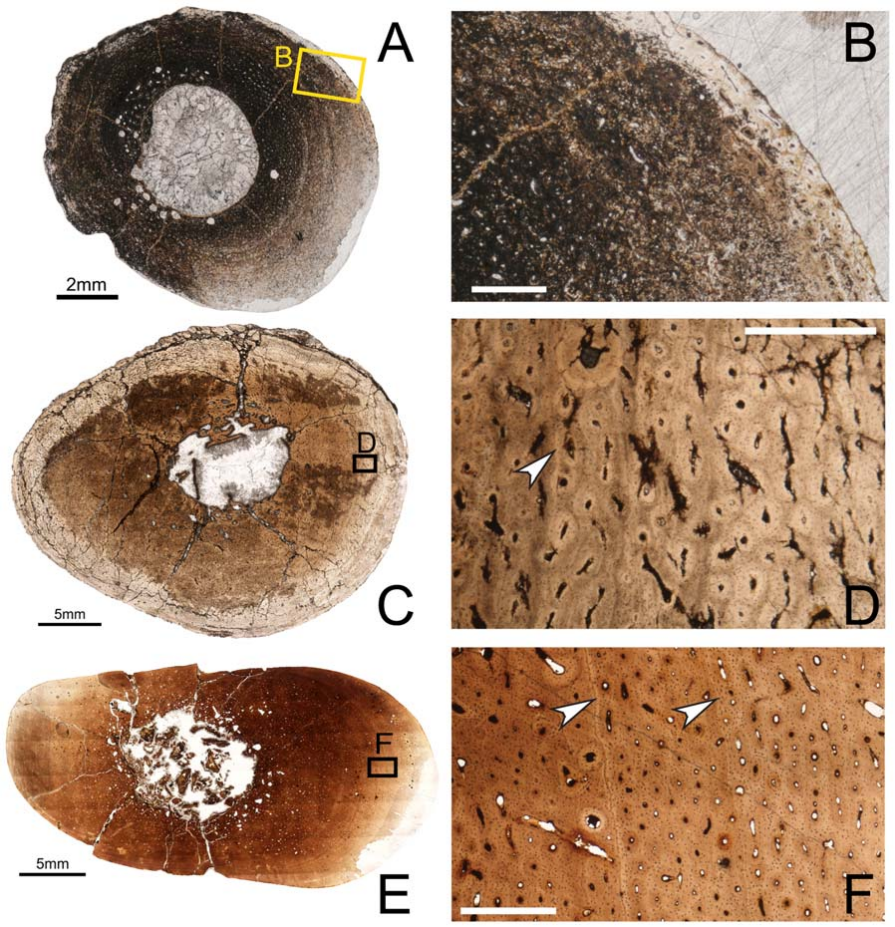
Osteohistology of the mid-diaphyseal fibula of *Tenontosaurus* in a juvenile (A,B) and two subadults (C–F). The cross-sectional geometry of the fibula changes from round to a flattened oval ontogenetically. **A.** Cross-section of OMNH 34785. This bone was invaded by bacteria before fossilization and thus much of the primary tissue is obscured. It is presented here in cross-section to illustrate vascular density and arrangement. **B.** Detail of the periosteal region of A. Longitudinal primary osteons and simple canals are visible in the periosteal region, but they do not show high levels of vascular connectivity.. The bone tissue is woven. **C.** Cross-section of OMNH 34783. This specimen was treated with oil before photography to increase light penetration, but this reduces the appearance of some thin, mineralized structures (LAGs, cement lines). **D.** Detail of the midcortex of C, showing the primary tissue of the midcortex. Most canals are longitudinal primary osteons which may show short anastomoses with one or two other canals. One secondary osteon is visible at the top of this image. A single LAG (arrow) is shown. **E.** Cross-section of OMNH 16563. **F.** Detail of the midcortex of E. As in D, canals are mostly longitudinal primary osteons. The weakly-woven bone is easier to ascertain in this image, based on the level of osteocyte disorganization. Scale bars: A = 2 mm; B,D,F = 0.5 mm; C,E = 5 mm.

The fibula of OMNH 34785 is nearly circular in cross-section (Figure 9A, 9B; MD: 7×9.5 mm; ACT: 2.25 mm), and the central medullary cavity is circular (MCD: 3×3 mm). As in the tibia and femur, bacterial invasion obscures much of the histology, but the vascular patterning is still clear. The fibula is well-vascularized throughout. The inner cortex shows a mixture of canal orientations. About half the canals are longitudinal, and half are short oblique, circumferential or reticular canals. The anastomoses between canals never connect more than three to five longitudinal canals. Some moderately large resorption rooms are in the inner cortex; these are about six to eight times the width of the vascular canals. There is also one relatively enormous resorption chamber extending two-thirds of the way into the cortex. In the mid- and outer cortex, the canals are predominantly longitudinally oriented. There is no preferred arrangement of the longitudinal canals in any region of the cortex. Where bone tissues can be observed (primarily on the endosteal and periosteal margins), they are woven with primary osteons. A small region preserves the endosteal margin, revealing two lamellae. However, calcite crystals have infilled the medullary cavity and destroyed much of the endosteal margin.

Subadult

Specimen examined: OMNH 16563, 34783

The cortex and medulla of the fibulae of OMNH 34783 (Figure 9C, 9D) and OMNH 16563 (Figure 9E, 9F) are both oval in cross-section (OMNH 16563: MD: 15.5×32 mm, ACT: 7 mm; MCD 8×11 mm; OMNH 34783: MD: 24×31 mm, ACT: 10.25 mm, MCD: 7.5×9 mm). The histology of both individuals is similar. In both cases, the cortex is thicker along the major axis (antero-postero axis) than along the minor axis (medio-lateral axis). Endosteally, a few intact trabeculae and resorption rooms are visible. These appear to have been formed by resorption of primary cortical tissues and are not separated from the rest of the cortex by lamellae. In OMNH 16563, the resorption rooms are lined with five to six lamellae. The centers of both bones are infilled with calcite crystals, and given the small size of this region, it is possible that a trabecular network spanned the center of the bone in life and that the fibula lacked a true medullary cavity.

Much of the cortex of the subadult fibula is densely remodeled with secondary osteons; no primary tissue can be observed anywhere in the inner cortex. Regionally this remodeling extends into the mid-cortex and even the outer cortex. Much of the inner cortex has undergone secondary remodeling, with secondary osteons overlapping previously-deposited ones. The primary bone tissue visible in the midcortex is woven bone with longitudinally oriented primary osteons, but the tissue is never as strongly woven as in the tibia or femur. The mid-cortex primarily exhibits longitudinally oriented canals, but a small amount of anastomosing does occur, resulting in very short circumferential, oblique, and radial canals. There is a trend of decreasing vascularity moving periosteally. In the outer cortex of OMHH 16563, there are a few longer radial canals, but most are oriented longitudinally. The diameter of the canals of the outer cortex is smaller compared to those of the mid-cortex. The bone tissue also becomes less woven moving periosteally. None of the primary bone tissue of the outermost cortex is truly woven bone, although thin bands of loosely parallel-fibered bone do occur between bands of more typical parallel-fibered bone in OMNH 34783. The canals of this region are longitudinally oriented simple canals in OMNH 34783, but in OMNH 16563, there are many primary osteons.

A single LAG is visible in the fibula of OMNH 34783 (compared to at least seven or eight in the tibia), and LAGs are visible in OMNH 16563 (2 are double LAGs). Given the extent of cortical remodeling and medullary cavity expansion in both individuals, it is likely that more LAGs have been obscured in both individuals. These sections were taken from the mid-diaphysis; there are no large muscle attachments at this point (these occur more distally in *Tenontosaurus*; see [11]) that may be influencing/stimulating the intense levels of bone remodeling. Because so much of the fibular histology is obscured in even younger individuals, this element probably should not be used for age estimations in this or related taxa.

## Discussion

### Ontogenetic variation in the bone histology of *Tenontosaurus*

#### Perinate

The bone histology of the perinate is similar to that of other perinatal archosaurs [16], [17], [47]. In the only perinatal bone examined in this study, the transition from previously deposited embryonic bone to perinatal bone is visible in the inner cortex. The embryonic tissue has large vascular spaces edged with a lamella, whereas the perinate tissue has no lamellae and the vascular spaces are much smaller. The cortex is entirely composed of strongly woven primary bone tissues, and shows only longitudinally oriented simple canals. Osteocytes are dense throughout the section and are organized around vascular canals. These vascular canals often open to the surface of the bone, giving the surface an irregular texture. No LAGs are visible at this growth stage.

#### Juvenile

In all long bones of this size class, the cortex is thicker than the diameter of the medullary cavity and is comprised of woven primary bone tissues with large, longitudinally oriented simple vascular canals that do not open to the surface of the bone. The vascular canals are circular or subelliptical in cross-section and smaller in diameter compared to those of the perinate. These canals are almost always arranged circumferentially in the cortex, and often show strong radial patterns as well, especially in the humerus and ulna. Osteocytes are dense throughout the cortex. These are organized around the vascular canals, but in the interstices between them, the osteocytes are randomly arranged and randomly oriented. In all specimens examined, the medullary cavity was filled by calcite crystals, and in most cases a thin band of avascular lamellar bone lines the edge of the marrow cavity, delimiting the endosteal boundary of the cortex. This band of lamellae suggests a pause in the expansion of the medullary cavity at this stage of growth. No LAGs or secondary osteons occur in any of the bones of this size class.

#### Subadult

Subadults always retain a thick cortex relative to the size of the medullary cavity, but at this growth stage, cortical bone tissues are removed as the medullary cavity expands. Thus, the records of embryonic, perinatal, and juvenile growth are absent in almost all subadult elements, with the possible exception of the femur of MOR 682. In most cases, the early record of subadult growth is probably missing as well, for the same reasons.

For all elements, the entire cortex is composed of well-vascularized woven bone. Except in the fibula, almost all of the visible cortical bone tissues are primary tissues vascularized by simple canals or (more commonly) primary osteons, but secondary osteons are visible endosteally in all subadult elements sampled. The amount and extent of secondary remodeling varies with the size (age) of the individual and by element. Younger individuals have secondary osteons only in the internalmost cortex, but these spread throughout the inner cortex and eventually into the mid-cortex in older animals. The tibia and femur show much less secondary remodeling compared to the humerus, ulna, and fibula.

All subadult elements show many longitudinally oriented canals throughout the cortex, and these are smaller in diameter compared to those of juveniles or perinates. More of these canals anastomose than in juveniles or perinates, and these anastomoses are longer (connect more longitudinal canals) in larger individuals. The anastomoses tend to be more circumferential in orientation in the tibia and femur, and more radial in the humerus and especially the ulna, but short connections occur in every direction in all elements. In larger subadults, true plexiform/laminar organization can be observed in the tibia, femur, and humerus. Interstitial osteocytes are dense throughout the subadult cortex and show no preferred arrangement relative to each other, nor a preferred orientation relative to the long axis of the bone.

All elements of subadult *Tenontosaurus* exhibited one or more LAGs. These often formed as “double LAGs”, i.e., two very closely-spaced LAGs that likely did not represent an entire year of growth between them. In many tibial sections, especially those from older individuals, the bone texture changed within each zone. In some elements, immediately following a LAG, a very thin band of parallel-fibered or weakly-woven bone tissue is present. This very quickly changes to woven bone, in which the collagen fibers were coarse and disorganized. The bone would become progressively less woven through the zone and leading up to the subsequent LAG. This suggests, for at least some elements, that the bone depositional rate decreased through the year, although lamellar-zonal bone (which is deposited much more slowly) was never observed in a subadult, and zonal width did not decrease dramatically in this size class.

#### Adult

Although only OMNH 62990 showed signs of growth truncation (i.e., an EFS in the outermost cortex), other signs of dramatically slowed growth in adult *Tenontosaurus* occur in all elements of FMNH PR 2261. Extensive remodeling (i.e., several generations of secondary osteons) of the inner, mid- and even outer cortex was observed in all elements. Additionally, the zonal cycles of decreasing growth rates truncating in a LAG observed in the larger subadults is more pronounced in adults. Moving periosteally through the cortex, the woven-to-less-woven bone transition becomes a weakly-woven-to-parallel-fibered pattern, and ultimately lamellar bone tissue is deposited in the outermost cortex. These transitions are accompanied by trends in decreasing vascularity (in terms of number of canals, not their size), decreasing vascular complexity (number and kinds of anastomoses), decreasing numbers of osteocytes, and decreasing zonal width. Together, these signals indicate several years of sustained slow growth rates in adult *Tenontosaurus* before an ultimate truncation of growth.

#### Summary of ontogenetic trends

Throughout early ontogeny and into subadulthood, *Tenontosaurus tilletti* is characterized by bone tissues associated with fast growth. The cortex of all of the major long bones exhibits woven bone tissue, high levels of vascularization, complex patterns of vascular connectivity and organization, and high osteocyte density. These histological characteristics suggest that *T. tilletti* maintained a rapid growth rate at least to the point of reproductive maturity [37]. However, at some point in the subadult stage, *Tenontosaurus* transitioned to a slower growth regime. These slower growth rates are initially reflected in the woven texture of the cortex of subadults, but in adults, further decreases in osteocyte and vascular density, smaller zonal widths, and ultimately the presence of an EFS suggest prolonged (several years) of slow growth rates before the termination of growth. These trends in bone histology strongly suggest asymptotic (“determinate”) skeletal growth for *Tenontosaurus*, consistent with what is observed in other dinosaurs [51]–[54] and in living archosaurs [46].

It is worth noting that the primary cortical tissues of *Tenontosaurus* meet the diagnostic criteria of fibro-lamellar bone (*sensu* [39]), during some, *but not all*, of its ontogeny. Perinatal and juvenile bone tissue, as well as the bone tissue of adults, does not always meet these criteria: some younger individuals lack the lamellar component of the fibro-lamellar complex, and the older individuals lack the woven component. Additionally, some subadult elements (e.g., the tibia of OMNH 16563) show a mixture of simple canals and primary osteons, whereas other subadults (e.g., the tibia of OMNH 34784) exhibit primary osteons exclusively. Therefore, the lamellar component of the fibro-lamellar complex is variably expressed. As it is only large juveniles and subadults that deposit true fibro-lamellar bone tissue, and among these the expression is variable, it is probably not appropriate to characterize the species as a whole as exhibiting this type of tissue. Depending on the age of the individual at death, most of the animal's life could be spent depositing other bone tissue types.

### Variation within the individual

The amount and rate of growth experienced by individual skeletal elements is not uniform across an individual's skeletons [36]. This is especially evident in subadult and adult specimens of *Tenontosaurus* because the number of LAGs is not consistent between elements of the same individual. Horner and colleagues [16], [36] noted this phenomenon in other ornithopod taxa. They found LAGs in some but not all elements of a single *Hypacrosaurus stebingeri* skeleton and also that the number of LAGs varied significantly by element in both *H. stebingeri* and in *Maiasaura peeblesorum*. In *Tenontosaurus*, there are several instances in which the number of LAGs do not match among elements: in OMNH 8137, the humerus has four LAGs but the tibia preserves six in OMNH 16563 the tibia preserves eight to nine LAGs and the fibula preserves six; in OMNH 34783 the tibia exhibits six to nine whereas the fibula shows only one; in OMNH 34784 the femur shows four and the tibia shows five; and in FMNH PR 2261 all elements preserve a different number of LAGS: the humerus has nine, the ulna seven, the femur ten, and the tibia at least eight. In fact, the only individuals in whom different elements preserve the same number of LAGs are juveniles that have no LAGs (OMNH 10144, OMNH 34785) and OMNH 2531, in which the humerus and ulna both have three LAGs.

The primary reason that different numbers of LAGs are preserved in *Tenontosaurus* seems to be that cortical remodeling and rates of medullary cavity expansion vary among different elements. The ulna, humerus, and fibula experience greater amounts of remodeling earlier in ontogeny compared to the femur and tibia and thus appear to be histologically older than other elements from the same skeleton. These LAG differences are not likely due to sampling biases. I controlled for sampling error in the OMNH and FMNH specimens by sampling a homologous area (the mid-diaphysis). The mid-diaphysis preserves the most primary bone tissue and consequently, the longest record of bone growth compared to other areas of the diaphysis [55]. Additionally, I traced each putative LAG around the circumferences of the bone and took several counts of the number of LAGs at various points around the circumference of the cortex.

For most individuals represented by more than one element, the tibia provided the highest LAG count of all elements sampled. In *Tenontosaurus*, the tibia is the least-remodeled of the limb bones from this taxon, except in PR 2261, in which it exhibited the highest levels of bone remodeling. This is consistent with the results of Horner et al. [16], who also found the tibia to be the most useful bone for sampling the ornithopod *M. peeblesorum*. However, Erickson et al. [51] found that in tyrannosaurids, the best elements for age determination were the fibula, pubis, and ribs. Those elements did experience cortical remodeling in life, but in tyrannosaurids preserved more primary cortex than the larger limb bones traditionally used in skeletochronological studies. It is likely that the ideal bone for sampling varies with phylogeny and morphology/biomechanics (e.g., the fibula is proportionately more substantial in *T. tilletti* relative to tyrannosaurids, and the difference in the extent of fibular remodeling may reflect differences in weight-bearing or other biomechanical influences in each taxon).

### Variation within and between populations of *Tenontosaurus*

Horner et al. [16] discussed four primary factors that influenced growth and how it is expressed in the bone tissues of dinosaurs: phylogeny, ontogeny, biomechanics, and environment. The first three factors have received much discussion in the dinosaur histology literature, but comparatively few studies have examined the effect of environment on dinosaurian bone histology, though it is known to have an effect on bone growth rate in extant tetrapods (e.g., [36], [41], [56]). This is likely because few studies have examined specimens sampled across a large paleogeographic range. Two exceptions are papers by Sander and Klein [57], [58], who studied the growth and osteohistology of the “prosauropod” *Plateosaurus engelhardti* from southern Germany and northern Switzerland. While both of those populations showed evidence of developmental plasticity in bone growth rate, the authors found no histological trends separating the Swiss and German populations.

*T. tilletti* is ideal for this type of comparison because populations are known from two paleogeographically distant locations—the Cloverly Formation of Wyoming and Montana, and the Antlers Formation of Oklahoma. During the Aptian-Albian, these formations would have been separated by 10–15 degrees of paleolatitude [59] and appear to have experienced differences in seasonality, average temperature, and average precipitation. Various studies have concluded that the paleoenvironment of the Cloverly would likely have been characterized by stronger wet-dry seasonality, cooler temperatures, and more precipitation than did the Antlers Formation [60]–[64]. These variables are known to cause variation in the osteohistology of extant vertebrates [36], [41], [56], [65]. If paleoenvironment differed with respect to factors that influence bone deposition rates, the histology may have been different for these two populations.

To control for differences in bone tissue type, ontogenetic stage, and skeletal element, I compared the primary cortical tissues of five subadult tibiae from the Antlers Formation (OMNH 2926, 8137, 16563, 58340, 63525) to three subadult tibiae from the Cloverly Formation (OMNH 10134, 34783, 34784). Tibiae were chosen for examination because they are numerically the most common bones sampled for this project and because they experienced the least amount of remodeling compared to other skeletal elements. Some size variation does exist in this subsample; specimens range in diameter (minor vs. major axis) from 34×40 mm to 47×57.5 mm. Though large differences in ontogeny do not exist in the subsample, slight differences in age might influence the results.

Subtle differences in cortical histology were observed between individuals, but no large trends differentiate the Antlers individuals from the Cloverly individuals. These differences relate to the ratio of simple canals to primary osteons, the extent of secondary remodeling, the number of LAGs preserved, and the pattern of disorganization in the woven bone between LAGs.

The smallest specimen, OMNH 63525, shows a mix of mostly simple canals with approximately twenty percent primary osteons, and very few secondary osteons restricted to the deep cortex. All other subadult tibiae examined showed mostly primary canals in the cortex, with some isolated primary canals and more extensive remodeling of the inner and midcortex. The extent and location of the remodeling was similar among all specimens except OMNH 63525 (very little) and OMNH 58340, which showed secondary remodeling reaching well into the midcortex. However, no consistent differences in vascular patterning or vascular arrangement could be observed among these individuals.

The number of LAGs varied with size, with larger individuals showing more LAGs. OMNH 63525 had five, and the largest specimens, OMNH 16563 and OMNH 34783, had as many as nine. This result is not unexpected, but the size difference between the largest and smallest subadult specimens is not great: only ∼1 cm along both the major and minor axes. Given that the amount/extent of remodeling is very similar for most of these individuals, it is likely that the number of LAGs lost to cortical remodeling and medullary cavity expansion is similar. This may represent a slowing of annual bone deposition rates not immediately apparent from qualitative examination of the bone histology. All specimens exhibited one or two double LAGs, but their distribution in the cortex (i.e., in which the LAG was a double LAG) shows no trends within or between populations.

The most interesting difference among these individuals was in the woven component of the bone, and how it changed cyclically between adjacent LAGs. In OMNH 2926, 10134, 58340, and 63525, the collagen fibers were consistent in their coarseness and in their disorganization across the zone. In OMNH 34784, the woven component of the bone became coarser and more disorganized moving toward the next ( = more periosteal) LAG. However, in OMNH 8137, 16563, and 34783, the opposite trend occurs, and bone gradually becomes more organized moving towards the next LAG. However, these differences cannot be linked to age/size or to geography. This suggests that *Tenontosaurus*, like *Plateosaurus* [57], [58] may have exhibited some amount of developmental plasticity in its growth.

Based on this subsample, I could find no evidence that differences in geography and/or paleoenvironment affected the appearance or deposition of bone histology in *T. tilletti*. It is possible that environment does not have a strong influence on dinosaurian bone histology, or that what appear to be two populations was actually one (e.g., if the species was migratory or the geographic gap in *Tenontosaurus* fossils is preservational and not real). Bone histology is conservative across vertebrates in general [39], [66], and the low sample size (n = 8) may preclude larger statements about histovariability across the species. It is also possible that population-level differences in histology occur in other elements, or at other ontogenetic stages, but I did not have the sample size to test these hypotheses. Though qualitative assessments of bone histology do not highlight differences between these two populations, it is possible that quantitative differences in growth curves may be observed.

### Osteohistological variation across Ornithopoda

Several previous studies have described the long bone osteohistology of ornithopod taxa. These include *Hypsilophodon* [44], [67], the “Proctor Lake ornithopod” [68], *Orodromeus* [17], [47], a “hypsilophodontid” from Dinosaur Cove/Flat Rocks, Victoria, Australia [44], [69], *Rhabdodon* [67], [70], [71], *Zalmoxes* [72], *Dryosaurus altus* [17], [47], *Dysalotosaurus lettowvorbecki* ([73] as “*Dryosaurus*” *lettowvorbecki*, [74]), *Valdosaurus* [67], *Camptosaurus* [17], *Iguanodon* [64], *Telmatosaurus* [72], *Hypacrosaurus* [19], [36], *Edmontosaurus* (“*Anatosaurus*” of [75]), and *Maiasaura* [16], [47], [76], [77]. The phylogenetic placement of most of these taxa (not all have been included in phylogenetic analyses) is indicated in Figure 1.

The aim of this section is not to present an exhaustive discussion of the osteohistology of ornithopod dinosaurs, but rather to outline the broader phylogenetic trajectory of the bone histology and implied growth rates within that clade, and to compare my observations for *Tenontosaurus* to those of other ornithopods both within and outside of Iguanodontia. I focus on the phylogenetic changes in ontogenetic bone histology moving from the base of Ornithopoda toward *Tenontosaurus*, in order to establish clearly that the ontogenetic osteohistology of *Tenontosaurus* is not unique, and then summarize the shared condition for the remainder of Iguanodontia.

*Orodromeus* is sister taxon to all other ornithopods sampled to date. Of these, it is also the smallest taxon (see Figure 1). Horner et al. [17] did not comment on the woven nature of the collagen fibers directly, but did indicate that the cortical tissues of juveniles were not lamellar in nature and that growth slowed to an EFS late in ontogeny. Horner et al. [17] observed that *Orodromeus* never achieved the complex vascular patterning seen in other ornithopods; rather, it produced mostly longitudinally oriented primary osteons throughout ontogeny. Small (young) individuals of *Tenontosaurus* and other larger ornithopods (e.g., *Maiasaura*) show much higher osteonal density compared to *Orodromeus*, implying a faster tissue deposition rate in the larger taxa at the ontogenetic point when they were same size as *Orodromeus*. Additionally, LAGs were visible in histologically juvenile individuals, and the largest adults only ever showed a couple of LAGs and an EFS. Secondary remodeling was limited to adults and apparently not extensive [17]. These histological signals suggest a pattern of slow growth relative to *Tenontosaurus*, but this may be explained in part by the difference in adult body size between the two species. However, unlike *Tenontosaurus*, *Orodromeus* did not experienced sustained slow growth.

A single section of *Hypsilophodon* was described by Reid [67] and figured in Chinsamy et al. [44], and no ontogenetic summary is available. However, it is clear that this animal grew faster than *Orodromeus* at any ontogenetic stage. Reid [67] reported woven bone (fibrolamellar tissue) throughout the entire cortex. Though the cortex is dominated by longitudinal primary osteons, most of these anastomose circumferentially (connecting two to five canals) or radially (connecting three to four canals). Histologically, the cortical bone looks very similar to the bone tissue of OMNH 63525, the smallest subadult in my sample. However, no LAGs are visible. It is clear that *Hypsilophodon* did not deposit LAGs as a juvenile, and that it reached faster tissue growth/deposition rates than *Orodromeus* even though it was not a substantially larger animal.

Other small “hypsilophodontids” (the Dinosaur Cove/Flat Rocks and Proctor Lake taxa) have not been placed phylogenetically but have been sampled ontogenetically. In an abstract, Winkler [68] described the femoral histology of a growth series of the Proctor Lake taxon. These individuals ranged in size from very small juveniles to subadults [68]. Specimens of all ages/sizes exhibited strongly woven bone, but vascular patterning and density were not described. Subadults and adults showed extensive secondary remodeling. None of the individuals sampled showed any LAGs or annuli. The lack of LAGs in even large subadults and adults close to growth truncation suggests several possibilities: 1) the Proctor Lake ornithopod grew longer than a single season but did not lay down LAGs because it never slowed its growth (as suggested for the Dinosaur Cove “hypsilophodontid” [44]; see below), 2) the taxon did lay down LAGs, but individual old enough in which to observe them has not yet been sampled, or 3) the taxon did not lay down LAGs because it finished growing within a single year. It is difficult to ascertain which of these hypotheses is most likely in the absence of a detailed histological analysis (and indeed, these hypotheses are not all mutually exclusive). The presence of secondary osteons in larger individuals is consistent with a more mature bone histology, and given the small adult body size of the taxon, it is reasonable that the taxon completed all or the majority of its growth within a single season. However, without further description and images of the histology, it is impossible to reject any of these hypotheses. Based on Winkler's description [68], the histology may have resembled that of *Hypsilophodon*.

Chinsamy et al. [44] described the femoral bone histology of an unnamed small-bodied “hypsilophodontid” from Dinosaur Cove and Flat Rocks localities, Victoria, Australia. The histological descriptions were recently revised and expanded [69]. Chinsamy et al. [44] and Woodward et al. [69] both noted a number of histological features in the Dinosaur Cove ornithopod suggesting that suggest initial rapid growth slowed through ontogeny in this taxon. Juveniles deposited woven bone but did not form annuli or LAGs. Woven bone continued to be deposited in individuals with up to three annuli/LAGs, after which poorly-vascularized parallel-fibered bone was deposited for a period of several years and only LAGs were deposited [69]. Though bone growth rate did not slow as early in ontogeny in *Tenontosaurus* (in terms of years) as in the Australian taxon, both ornithopods share a pattern of initial rapid growth followed by sustained growth. However, the Dinosaur Cove/Flat Rocks taxon appears to have undergone this transition in early subadulthood, whereas it is only visible in late subadults and adults of *Tenontosaurus*. However, this may reflect the small size of the Australian taxon (∼315 mm adult femoral length [69]).

The rhabdodontids are sister taxon to all other iguanodontians. The histology of subadult and adult *Zalmoxes robustus* and *Zalmoxes shqiperorum* was described by Benton et al. [72]. The authors found strongly woven primary bone deposited through ontogeny, with a reticular arrangement of primary osteons that later graded into a circumferential pattern and eventually to longitudinal canals in the oldest adults. From the figures in Benton et al. [72], it is clear that canal density was much lower in more external zones. Benton and colleagues also noted that remodeling of the cortex did not begin until the late subadult stage, but was dense in much of the adult cortex. Subadults and adults exhibited numerous LAGs (7 in the largest *Z. robustus* and 13 in the largest *Z. shqiperorum*). An EFS could not be confirmed because of surface abrasion, but the authors diagnosed adults based on the amount of remodeling and the high number of LAGs in the largest specimens. However, although the LAG count of *Z. shqiperorum* is higher than in other ornithopods discussed so far, the LAG count in *Z. robustus* is the same as that of the largest Dinosaur Cove/Flat Rocks individual, which is of comparable size. The same pattern of initial fast growth followed by several years of sustained slower growth also seems to have characterized both species of *Zalmoxes*.

Limb material of *Rhabdodon* was described by Nopcsa [70] and by Reid [71], [75]. Although the two earlier papers describe the long bone histology of *Rhabdodon* as “lamellar-zonal” (lamellar bone broken into annual zones of deposition by LAGs), it is clear from Reid [71] that this is only correct in the outermost cortex (see especially [71]: Figure 24). Reid re-examined the histology of an adult femur and two tibiae were re-examined [71]. In those specimens, the inner and midcortex could not have been lamellar in nature; the midcortex show disorganized osteocytes more consistent with parallel-fibered or woven bone tissue. The vascular complexity is never high; most canals are longitudinal primary osteons (in the midcortex) or simple canals (in the outer cortex) and do not anastomose with more than one other canal. Secondary remodeling of the inner cortex was dense in the adult figured in that paper. Reid [71] estimated based on LAGs that the adult grew rapidly for nine or ten years, followed by slow growth for ten more years, and noted an EFS comprising four additional years. From the image, it appears that at least four of the LAGs in the “slow growth” phase are double LAGs using my diagnostic criteria. Regardless, the same pattern of sustained slowed growth as an adult clearly occurred in *Rhabdodon*. However, from the lack of vascular patterning it is clear that *Rhabdodon* was growing relatively slower than *Tenontosaurus*, *Zalmoxes*, and the Dinosaur Cove/Flat Rocks taxon during even the rapid phase of its growth.

The pattern of growth seen in *Tenontosaurus* (this study, [11]) is consistent with that of its successive outgroups except *Orodromeus*: initial rapid growth, represented by woven bone, dense longitudinal canals connected by long circumferential anastomoses, and wide zones between annuli and/or LAGs. This pattern gives way to narrower zones showing lower vascular density and complexity and more organized collagen fibers, beginning in the subadult stage. Secondary remodeling of the innermost cortex also begins in the subadult stage. This pattern is sustained for several years until skeletal maturity is reached, reflected by a transition in the outermost cortex to avascular lamellar bone and often an EFS. This is coupled with dense secondary remodeling of the inner and midcortex.

The rest of Iguanodontia shows a markedly different growth pattern. These taxa (e.g., *Dryosaurus*, *Dysalotosaurus*, *Valdosaurus*, *Camptosaurus*, *Iguanodon*, *Telmatosaurus*, *Maiasaura*, and *Edmontosaurus*) are characterized by more complex vascular patterning (e.g., reticular canals or more extensive circumferential canals) beginning much earlier in ontogeny (e.g., *Dryosaurus* [17], *Dysalotosaurus* [74], *Valdosaurus* [67], *Telmatosaurus* [72], *Maiasaura* [16]). They also deposit strongly woven bone in consistently widely-spaced zones well into the subadult or even adult growth stage, and remodeling of the inner cortex begins much earlier in ontogeny [e.g., *Telmatosaurus* [72] or *Maiasaura* [16]). These histological traits are consistent with faster growth rates in a relative sense [78]–[80], but the bones of most of these taxa are larger at each ontogenetic stage compared to those of *Tenontosaurus*, so they were growing at absolutely faster rates as well [78]. Most taxa also seem to lay down fewer LAGs (e.g., *Dryosaurus* [17], *Dysalotosaurus* [73], [74], or *Maiasaura* [16]) compared to *Tenontosaurus*, which may reflect a shorter growth duration (in terms of number of years) or such fast growth in early years that LAGs are not deposited [36], [73], [74]. The transition to slowed growth occurs late in ontogeny (late subadult stage) and is characterized by narrowing zonal widths, but not changes in bone tissue type. That is, the bone tissue in those narrow zones is virtually identical in collagen disorganization, vascular density, and vascular patterning compared to more endosteal primary cortical tissues (e.g., *Dryosaurus* [17], *Dysalotosaurus* [74], *Telmatosaurus* [72], or *Maiasaura* [16]). For the ornithopod taxa in which an EFS has been observed (*Hypacrosaurus* [19] and *Maiasaura* [16]), the transition between woven bone to lamellar bone is abrupt.

Several factors complicate osteohistological interpretations and comparisons of these taxa. Until around the year 2000, access to complete, diagnostic specimens for histological sampling was not routinely granted by museum curators, and researchers were limited in the quality of specimen available for sectioning. Even when complete elements were used, apomorphies and/or unique combinations of characters were not used to identify specimens in most of these studies, so some taxonomic identifications may not be correct. The exact sampling location and ontogenetic ages of sampled specimens were not reported in some studies, so the homology of the sampling location cannot be confirmed (although most studies since 2000 do report sampling the midshaft). Finally, ornithopod body size is not independent of phylogeny; all taxa outside of Iguanodontia are similar to *Tenontosaurus* in size or smaller, and almost all other Iguanodontians are similar to *Tenontosaurus* in size or larger [81]. Moving closer to Hadrosauria phylogenetically, adult body size increases (Figure 1). Adult body mass influences both growth rate and the bone histology it reflects; all else being equal, small animals grow at absolutely slower rates and show a “slower” signal of growth in their bone histology [39], [54], [78]–[80].

These caveats notwithstanding, the examination of osteohistology in phylogenetic context suggests a distinct change in the growth biology of ornithopods near (but not at) the base of Iguanodontia. One growth syndrome (sustained slow growth/bone depositional rates for several years late in ontogeny) is typified by *Tenontosaurus* and its successive outgroups. All other iguanodontians share a similar syndrome of extended rapid growth and bone depositional rates, which transition to tissues associated with the end of active skeletal growth abruptly. This suggests a physiological shift in bone growth, and this shift is phylogenetically coincident with a shift in body size and an increase in taxic diversity [14], [82] (Figure 1). From the data at hand, it is not clear whether this physiological shift in growth rate permitted the increase in body size seen in more derived iguanodontians (i.e., a key innovation of sorts) or merely reflects it (because body size correlates with growth rate in an absolute sense). A trend of increasing body size is seen across the entire clade, but the largest sizes and the great diversity of ornithopods are not attained until after this transition in growth occurred. *Tenontosaurus* is “medium-sized” for the clade, but it may be the largest size an ornithopod could grow under the basal growth syndrome.

Unfortunately, our ability to address the nature of this transition is currently limited for taxonomic sampling reasons. The phylogenetic distribution of the ornithopod taxa sampled for growth studies does not reflect the actual diversity of Ornithopoda. Many more of the sampled ornithopods fall outside Ankylopollexia than within it, but Ankylopollexia contains the vast majority of ornithopod taxa [82]. The key to determining how growth rate and size are linked in Ornithopoda, as well as how these changes vary ontogenetically, will require hadrosaurs and non-hadrosauroid ankylopollexians to be sampled much more densely.

### Conclusions

The osteohistology of *Tenontosaurus tilletti* suggests that this species grew rapidly as a perinate, juvenile, and early subadult; laying down well-vascularized woven bone tissue during each of these stages. Beginning in subadulthood, *Tenontosaurus* changed its growth regime and began depositing parallel-fibered bone tissue in much narrower zones. Similar to its successive outgroups, but markedly different from all other iguanodontians, *Tenontosaurus* experienced a protracted period of slow growth as skeletal maturity approached. Evidence of skeletal senescence is observed only in the two largest individuals sampled, although the histology of slightly smaller specimens is consistent with substantially slowed growth during the last few years before skeletal maturity. As in other dinosaurs, bone tissue varied among elements with regards to their relative remodeling rates and LAG counts. However, comparison of the tibial histology of subadults from two geographically-distant collection areas (the Antlers and Cloverly formations) showed surprisingly little variation in tissue morphology, despite known differences in paleoenvironment between these areas. When compared to other ornithopods in phylogenetic context, the bone histology suggests that *Tenontosaurus* retained the plesiomorphic condition (fast growth early in life, followed by an extended period of much slower growth and bone deposition rates later in life). This is much different than was has been observed in all other iguanodontians, who show no evidence of protracted slow growth later in life. However, the ontogenetic osteohistology of iguanodontians is much more poorly sampled relative to non-iguanodontian ornithopods. In order to understand how and when the growth pattern observed in hadrosaurs evolved, greater histological sampling of ankylopollexians is needed.
